# Microbial Signatures in Deep CO_2_-Saturated Miocene Sediments of the Active Hartoušov Mofette System (NW Czech Republic)

**DOI:** 10.3389/fmicb.2020.543260

**Published:** 2020-12-14

**Authors:** Qi Liu, Karsten Adler, Daniel Lipus, Horst Kämpf, Robert Bussert, Birgit Plessen, Hans-Martin Schulz, Patryk Krauze, Fabian Horn, Dirk Wagner, Kai Mangelsdorf, Mashal Alawi

**Affiliations:** ^1^Section Geomicrobiology, GFZ German Research Centre for Geosciences, Potsdam, Germany; ^2^Institute of Geosciences, University of Potsdam, Potsdam, Germany; ^3^Section Organic Geochemistry, GFZ German Research Centre for Geosciences, Potsdam, Germany; ^4^Section Applied Geochemistry, Institute of Applied Geosciences, Technische Universität Berlin, Berlin, Germany; ^5^Section Climate Dynamics and Landscape Evolution, GFZ German Research Centre for Geosciences, Potsdam, Germany

**Keywords:** geo-bio interaction, CO_2_, mofette systems, Eger Rift, microbial lipid biomarker, microbial diversity, deep biosphere, saline groundwater

## Abstract

The Hartoušov mofette system is a natural CO_2_ degassing site in the central Cheb Basin (Eger Rift, Central Europe). In early 2016 a 108 m deep core was obtained from this system to investigate the impact of ascending mantle-derived CO_2_ on indigenous deep microbial communities and their surrounding life habitat. During drilling, a CO_2_ blow out occurred at a depth of 78.5 meter below surface (mbs) suggesting a CO_2_ reservoir associated with a deep low-permeable CO_2_-saturated saline aquifer at the transition from Early Miocene terrestrial to lacustrine sediments. Past microbial communities were investigated by hopanoids and glycerol dialkyl glycerol tetraethers (GDGTs) reflecting the environmental conditions during the time of deposition rather than showing a signal of the current deep biosphere. The composition and distribution of the deep microbial community potentially stimulated by the upward migration of CO_2_ starting during Mid Pleistocene time was investigated by intact polar lipids (IPLs), quantitative polymerase chain reaction (qPCR), and deoxyribonucleic acid (DNA) analysis. The deep biosphere is characterized by microorganisms that are linked to the distribution and migration of the ascending CO_2_-saturated groundwater and the availability of organic matter instead of being linked to single lithological units of the investigated rock profile. Our findings revealed high relative abundances of common soil and water bacteria, in particular the facultative, anaerobic and potential iron-oxidizing *Acidovorax* and other members of the family *Comamonadaceae* across the whole recovered core. The results also highlighted the frequent detection of the putative sulfate-oxidizing and CO_2_-fixating genus *Sulfuricurvum* at certain depths. A set of new IPLs are suggested to be indicative for microorganisms associated to CO_2_ accumulation in the mofette system.

## Introduction

**Figure 1:**
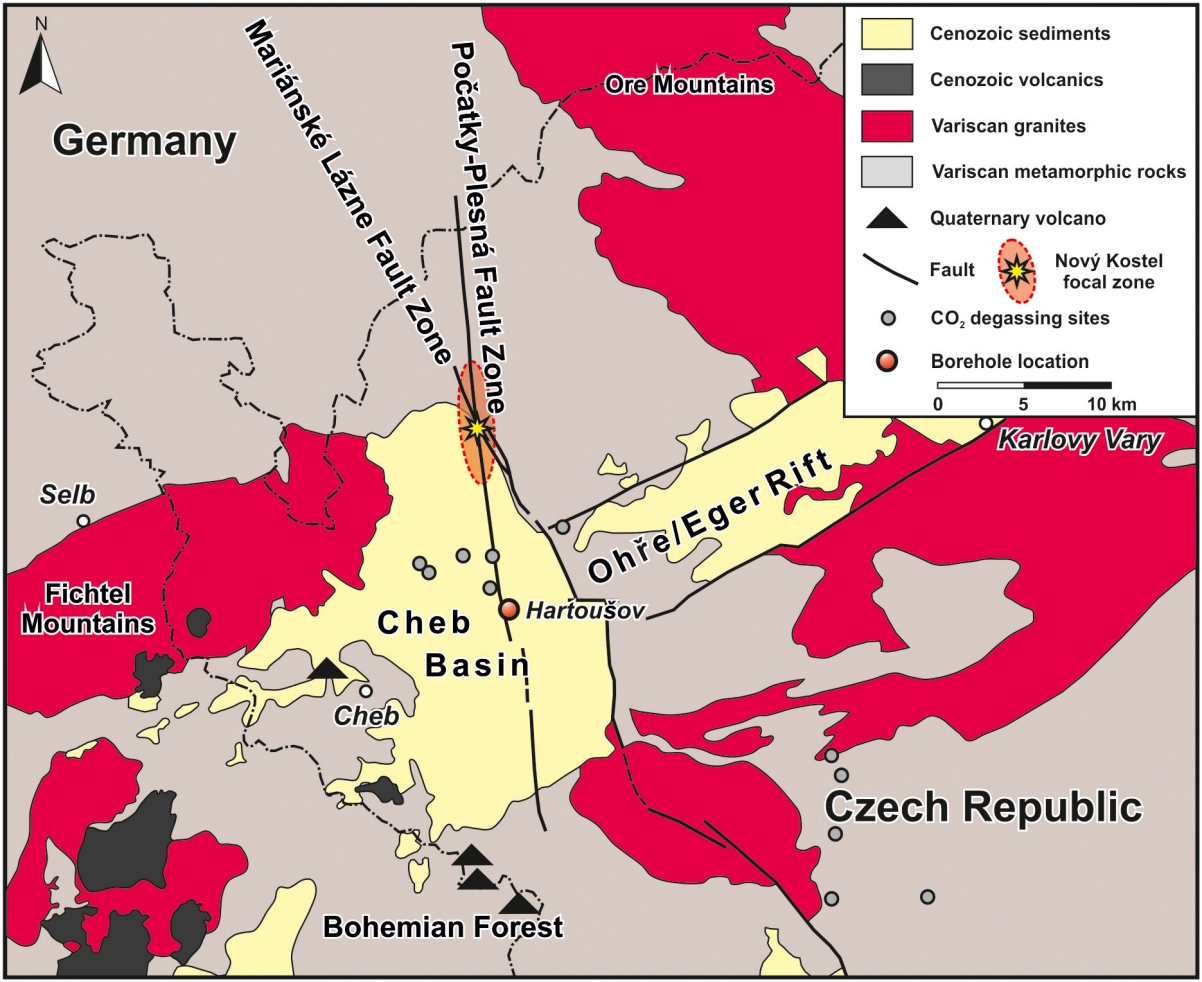
Map of the study site in NW Czech Republic showing the position of the Hartoušov mofette system in the center of the Cheb Basin that evolved at the intersection of the Eger Rift and Regensburg-Leipzig-Rostock Fault Zone, here represented by the Mariánské Lázně and the Počatky-Plesná Fault Zones (modified after Liu et al., 2018).

The Hartoušov mofette system is located in the center of the Cheb Basin (Eger Rift) at the central part of the Počatky-Plesná Fault Zone (PPZ) (Bankwitz et al., 2003a,b; Flechsig et al., 2008, 2010; Nickschick et al., 2019; Figure 1). The region is known for periodically occurring earthquake swarms and widely distributed natural cold gas exhalation systems in form of mofette sites and mineral water springs releasing CO_2_-rich gas into the atmosphere (Fischer et al., 2014). The CO_2_ originates from active magma chambers at the crust-mantle boundary and from lithospheric mantle depths of about 65 km (Heuer et al., 2006; Bräuer et al., 2009). The CO_2_ preferentially migrates to the surface as component of supercritical fluids in the lower crust or either dissolved in water or as a free gas phase along deep-seated faults in the upper crust (Weinlich et al., 1999; Weise et al., 2001; Bräuer et al., 2011; Kämpf et al., 2019). The PPZ started to develop at the boundary from Mid to Late Pleistocene (Bankwitz et al., 2003a,b). CO_2_-rich nephelinitic magma (Seifert and Kämpf, 1994; Geissler et al., 2007; Brandl et al., 2015) erupted in the Mid Pleistocene (Mrlina et al., 2007; Rohrmüller et al., 2018; Krmíček et al., 2020; Lied et al., 2020). Age determinations of the hydrothermal activity of Karlovy Vary spa with travertine deposits go back to 0.23 Ma (Vylita et al., 2007). The age of the Hartoušov mofette system is unknown so far, but in this study assigned to the onset of PPZ development and the occurrence of volcanism. Previous investigations from sediments of the upper 9 m at the Hartoušov mofette system revealed that ascending CO_2_-containing fluids cause sediment fluidization, hydrofracturing, and geochemical alterations e.g., sediment bleaching, mobilization of metals and the preservation of organic matter (Flechsig et al., 2008; Rennert et al., 2011; Mehlhorn et al., 2016, 2018; Rennert and Pfanz, 2016; Bussert et al., 2017; Liu et al., 2018). At the surface, CO_2_ exhalation occurs in form of diffuse degassing structures (DDS, namely dry mofettes) and localized water filled, pool-like structures (wet mofettes) (Flechsig et al., 2008; Kämpf et al., 2013, 2019; Nickschick et al., 2015, 2017).

Dry mofette areas display high CO_2_ soil gas concentrations, low soil pH, accelerated silicate weathering, leaching of base cations, anomalous vegetation patterns, low taxonomic, and functional biodiversity of soil biota and a limited microbial degradation of soil organic matter (SOM) (Rennert et al., 2011; Hohberg et al., 2015; Beulig et al., 2016; Rennert and Pfanz, 2016; Kämpf et al., 2019). In comparison to reference sites, the microbial community differs in composition and is dominated by anaerobic chemolithoautotrophic microorganisms, e.g., acidophilic, methanogenic, and sulfur-cycling organisms (Beulig et al., 2015, 2016; Liu et al., 2018). Additionally, a higher microbial abundance was observed even in deeper parts of the sedimentological profile (Liu et al., 2018) and estimates for microbial fixation of ascending geogenic CO_2_ reach up to 27% of the total SOM (Nowak et al., 2015). Similar characteristics were also found at other mofette sites, e.g., the Laacher See in Germany (Krüger et al., 2009; Frerichs et al., 2013), the Latera caldera in Italy (Oppermann et al., 2010), and the Stavešinci mofette in Slovenia (Šibanc et al., 2014).

Hydrogeochemical investigations of waters from wet mofettes and mineral springs in the Cheb Basin by Krauze et al. (2017) and in the adjacent areas (Wagner et al., 2007; Schuessler et al., 2016) unraveled different water sources, with surface water at some locations being co-sourced by a deep saline aquifer. The microbial communities in all of these CO_2_-influenced waters were generally dominated by chemolithoautotrophic microorganisms (iron- and sulfur-cycling organisms) and methanogenic archaea. Similar to dry mofettes, the microbial degradation of complex dissolved organic carbon (DOC) is also restricted in these anaerobic environments (Krauze et al., 2017), suggesting that upstreaming CO_2_ is also one of the main carbon sources for microorganisms. The connection to a deep saline aquifer at some mofettes (e.g., Bublak, ~1.5 km NNE of the Hartoušov mofette) was indicated by the occurrence of specific microorganisms from the deep subsurface biosphere and marine paleoenvironments pointing to a widespread deep saline aquifer as a general deep microbial habitat in this region (Krauze et al., 2017).

In addition, other deep biosphere habitats may exist that are associated with CO_2_ reservoirs in geological trapping structures, as indicated by an increase in gas flow rates after swarm earthquakes pointing to a gas release after seismically induced fracking of sealing layers (Sandig et al., 2014; Sauer et al., 2014; Schuessler et al., 2016; Fischer et al., 2017). Additionally, Kämpf and Bankwitz (2005) described dm- to m-sized cavities in sediments of the nearby Nová Ves II open-cast mine at 50 mbs (meter below surface), which developed along fluid migration pathways. This suggests the presence of restricted gas-filled cavities, which may function as distinct habitats for the deep biosphere. An important indication for a CO_2_-related deep biosphere was recognized by Bräuer et al. (2005) after a swarm earthquake activity in 2000. They detected an increase in methane concentrations at the Wettin spring (Bad Brambach, Germany) about 20 km north of the Hartoušov mofette system, where a significant decrease of δ^13^C_methane_ was attributed to microbial methane production from magmatic CO_2_ and pre- or co-seismically released hydrogen from the granitic basement. After a swarm earthquake event in 2011, higher methane concentrations were also detected at the Bublak mofette (Bräuer et al., 2018).

These previous investigations show that ascending geogenic CO_2_-containing fluids locally alter the sedimentary overburden and thus change the environmental conditions for microbial life. Additionally, there is evidence of subsurface structures that may host CO_2_-influenced deep microbial habitats, which could function as deep microbial hotspots. However, studies investigating the potential for CO_2_-related deep microbial life in the Cheb Basin and the Eger Rift are still missing. Thus, in early 2016 the German Research Centre for Geosciences (GFZ) drilled a 108.5 m deep borehole as a test case for the International Continental Scientific Drilling Program (ICDP) project “*Drilling the Eger Rift*” (Dahm et al., 2013). The borehole was positioned in the Hartoušov mofette system (HJB-1) (50°07′58″N, 12°27′46″E) and described in detail by Bussert et al. (2017). During drilling, CO_2_-rich sediments were recovered between 71 and 81 mbs. At a depth of 78.5 mbs a CO_2_ blow out occurred, suggesting the presence of a subsurface CO_2_ accumulation. This CO_2_ reservoir is associated to a basal low-permeable CO_2_-saturated and saline aquifer (1,892 mg L^−1^ of free dissolved CO_2_) that occurs between 79 and 85 mbs at the transition from Early Miocene terrestrial to overlying lacustrine sediments. Hydrogeochemically, the aquifer is characterized by a Na-Ca-HCO_3_-SO_4_-type water with a high Fe content of up to 13.7 mg L^−1^ and a pH of 6.4 (Bussert et al., 2017). Due to the potential of the CO_2_-saturated aquifer to host a very specialized microbial community we focussed on the core interval between 65 and 95 mbs. Our aim was to identify the impact of mantle-derived CO_2_ on deep microbial communities and to find out whether the low-permeable CO_2_-saturated and saline aquifer might act as a hotspot for present deep microbial life. The methodological approach to characterize the microbial community included lipid biomarker analysis of past and living microbial biomass (hopanoids, GDGTs and intact polar lipids) as well as DNA analysis such as quantitative Polymerase Chain Reaction (qPCR) and Illumina 16S rRNA gene amplicon sequencing. Furthermore, the microbial signals were compared to lithological background information and sedimentological bulk parameters.

## Methods

### Drilling, Coring, and Pump Test

A detailed description of the field work including drilling, coring and a pump test was published by Bussert et al. (2017). The drilling was performed with a Drillmec G-25 device installed on a Tatra 815 drilling lorry which discovered core material in PVC liners with a length of 3 m and a diameter of 0.1 m. The drilling mud consisted of homogeneously blended pure bentonite. In order to monitor potential drill mud contamination of the retrieved core material, sodium fluorescein was added to the drill mud with a concentration of 5 mg L^−1^ (Supplementary Figure 1D) according to Pellizzari et al. (2013). Subsamples for further analysis were taken about every 0.5 m and stored in gasbags flushed with nitrogen at −80°C directly after core recovery in the field. After the drilling campaign a 24 h pump test within the deep low-permeable CO_2_-saturated saline aquifer was performed. The groundwater was filtered, the obtained water samples geochemically analyzed and the obtained filters stored at −20°C, respectively.

### Sample Processing and Contamination Control

The initial lithological description of the sample material and the drill mud contamination control were performed in the lab. The frozen core segments were stored over night at 5°C to initiate thawing of the external sample layer and to avoid fluid migration from the rim to the center of the samples. The thawed rim (~1 cm) was removed (inner coring), the still frozen inner core described (e.g., Supplementary Figure 1), material from the removed rim (outer rim) tested in triplicates for fluorescein (Pellizzari et al., 2013) and the samples again stored at −80°C. To ensure that the samples are not contaminated by external DNA the inner coring technique was repeated in a clean bench (Thermo Scientific, Waltham, USA). The removed material and the outside of the inner core were again tested in triplicates for fluorescein (inner rim). Inner core samples (sample) exceeding the background fluorescence were excluded from further analysis (Supplementary Figure 1). The fluorescein concentration was measured with a CLARIO star® plate reader (BMG LABTECH GmbH, Ortenberg, Germany). The background fluorescence signal was obtained from samples of a shallow drilling campaign (3 m) drilled in 2015 adjacent to our study side without the application of drill mud and fluorescein (Liu et al., 2018).

### Bulk Carbon and Nitrogen Analyses

Total carbon (TC), total organic carbon (TOC), total nitrogen (TN), and the bulk δ^13^C_org_ were all analyzed with the same equipment consisting of a NC2500 Carlo Erba elemental analyser coupled with a ConFlo_III interface on a DELTAplusXL isotope ratio mass spectrometer (IRMS) (Thermo Fischer Scientific). Prior to analysis the sample material was freeze-dried, powdered and homogenized. In order to determine the TC and TN ~25 mg of sample material was loaded into tin capsules and the content was calibrated against acetanilide. For investigation of TOC and bulk δ^13^C_org_ the carbonate content was removed using *in situ* decalcification. Therefore, depending on the TOC content, 3–10 mg sample material were loaded into Ag-capsules and decalcified by drops of 3% HCl followed by 20% HCl and heated for 3 h at 75°C. The calibration was performed using elemental urea and certified isotope standards (USGS24, IAEA-CH-7) and proofed with an internal soil reference sample (Boden3, HEKATECH). All isotope compositions are given relative to the VPDB (Vienna Pee Dee Belemnite) standard in the conventional delta notation. The total inorganic carbon (TIC) was calculated by subtraction of TOC from TC.

### Lipid Biomarker Extraction and Chromatographic Column Separation

The freeze-dried, powdered and homogenized sediment samples (about 80 g) were extracted with a modified extraction method after Bligh and Dyer (1959) using methanol:dichloromethane (DCM):ammonium acetate buffer (pH 7.5) (2:1:0.8) as initial extraction solvent mixture. The sample material was admixed with the extraction solvent (4x sample mass in mL, ~320 mL), stirred with a flow-blending rod for 5 min and afterwards centrifuged for 10 min with 2,500 rpm. The supernatant was transferred to a separation funnel and the remaining sample 2 times re-extracted in an ultrasonic bath for 10 min, followed by centrifugation and transfer of the supernatant into the separation funnel. To achieve phase separation, the solvent ratio in the separation funnel was changed to 1:1:0.9 (methanol:DCM:ammonium acetate buffer). Afterwards the organic phase containing the lipid extract was collected in a turbovap glas and the solvent removed (TurboVap 500). Each fifth sample was a blank. After extraction 5α-Androstane and deuterium-labeled phosphatidylcholine (PC_d54_ = 1,2-dimyristoyl-d54-sn-glycero-3-phosphocholine) were added as standards for compound quantification in the aliphatic and intact polar lipid fractions, respectively. The obtained total extracts were chromatographically separated into a low polar lipid (20 mL chloroform), free fatty acid (50 mL methyl formiate with 0.025% glacial acetic acid), glycolipid (20 mL acetone), and intact polar lipid (IPLs, 25 mL methanol) fraction using two glass syringe columns filled with dried pure silica (1 g silica gel 63–200 μm, dried at 110°C for 2 h) and Florisil (1 g magnesium silica gel 150–250 μm) with the silica column on top of the Florisil column. The IPL fraction was only eluted from the silica column (Zink and Mangelsdorf, 2004). To improve IPL recovery the silica column was eluted with 25 mL methanol:water (60:40) for a second time. Phase separation was conducted as described above. Finally the IPL fractions were combined and the solvent removed. Afterwards, the IPL fraction was split into two halves: one for the direct detection of IPLs and one for the detection of polar lipid fatty acids (PLFAs) after saponification (Müller et al., 1993).

After removal of asphaltenes the low polar lipid fraction was further subdivided by Medium Pressure Liquid Chromatography (MPLC) into an aliphatic, aromatic, and Nitrogen-Sulfur-Oxygen-containing compound (NSO) fraction (Radke et al., 1980). The aliphatic fraction was analyzed for hopanoids and the NSO fraction for glycerol dialkyl glycerol tetraethers (GDGTs). GDGTs have been quantified with regard to an external archaeol standard.

### Determination of the Lipid Biomarkers

Analysis of IPLs was performed on a Thermo Scientific Ultimate 3000 RS Ultra high performance liquid chromatograph (UHPLC) coupled to a Q Exactive Plus Orbitrap mass spectrometer (MS) with a heated electrospray (H-ESI II) probe. Samples were separated with a LiChrospher 100 diol column (2 × 125 mm, 5 μm; CS-Chromatographie Service) equipped with a pre-column filter. The eluents used for compound separation were (A) *n*-hexane:isopropanol:formic acid:ammonia (25% in water) 79:20:1.2:0.04 v/v and (B) isopropanol:water:formic acid:ammonia (25% in water) 88:10:1.2:0.04 v/v (solvent gradients: 1 min 100% A, linear increase of B to 65% within 20 and 40 min for reconditioning). The flow rate was set to 0.35 mL/min (modified after Rütters et al., 2001). ESI source conditions were as follows: spray voltage −2.2 kV; capillary temperature 300°C; nitrogen sheath gas at 49 and auxiliary gas at 12 arbitrary units at a temperature of 419°C, S-Lens 65 V. The obtained data were acquired in negative and positive ion mode with dependent MS/MS acquisition at ranges of m/z 400–2,000. The full scan and fragment spectra were collected at a resolution of 280,000 and 70,000 (at m/z 200), respectively.

The aliphatic fraction and PLFAs were determined on a Thermo Trace GC Ultra equipped with a Thermo PTV injection system and a SGE BPX5 fused silica capillary column (50 m length, 0.22 mm ID, 0.25 μm film thickness) coupled to a Thermo Trace DSQ Quadrupole MS. Helium was used as carrier gas. The temperature of the GC oven was programmed from 50°C (hold 1 min) to 310°C at a rate of 3°C min^−1^, followed by an isothermal phase of 30 min. The injector temperature was programmed from 50 to 300°C at a rate of 10°C s^−1^. The MS was operated in electron impact ionization mode (EI) at 70 eV. Full scan mass spectra for compound identification were recorded from m/z 50 to 600 at a scan rate of 1.5 scans s^−1^.

GDGT analysis was conducted on a Shimadzu LC10AD HPLC instrument coupled to a Finnigan Triple Stage Quadrupole (TSQ) 7000 MS with an atmospheric pressure chemical ionization (APCI) interface. Samples were separated at 30°C with a Prevail Cyano column (2.1 × 150 mm, 3 μm; Alltech) equipped with a pre-column filter. The mobile phase consisted of (A) *n*-hexane and (B) isopropanol and compound separation was achieved using the following solvent gradients: 5 min 99% A and 1% B, linear gradient to 1.8% B within 40 min, increase to 10% B within 1 min and holding time for 5 min to clean the column, back to initial solvent conditions within 1 min and 16 min for column equilibration (Schouten et al., 2007). The flow rate was set to 200 μL min^−1^. The APCI adjustments were: corona current 5 μA giving a voltage of around 5 kV, vaporizer temperature 350°C, capillary temperature 200°C and nitrogen sheath gas at 60 psi (no auxiliary gas). Mass spectra were generated by selected ion monitoring in the positive ion mode for the masses 1295.0, 1302.1, 1049.5, 1035.5, 1021.5, and 654.2 each with a width of 7 amu (to also obtain neighboring masses) representing major core GDGTs at a scan rate of 0.33 s.

Compound specific δ^13^C values of the aliphatic fraction (hopanoids) were determined with a GC-isotope ratio monitoring (IR)-MS system consisting of an Agilent 7890 GC (USA) connected with an open split GC-C/TCIII-Interface for compound-specific carbon and hydrogen isotope analysis to a Delta V Plus IRMS (Thermo Fischer Scientific, Germany). The GC-separated organic substances were oxidized to CO_2_ in a combustion furnace at a temperature of 940°C on a CuO/Ni/Pt catalyst. CO_2_ was transferred to the mass spectrometer to determine carbon isotope ratios. Three microliter of the aliphatic fraction were injected with a split ratio of 1:2 and an initial temperature of 230°C to a programmable temperature vaporization inlet (PTV, Agilent Technology, USA). The injector was heated to 300°C with a heating rate of 12°C s^−1^. The separation of the aliphatic fractions was attained by a fused silica capillary column (HP Ultra 1, 50 m × 0.2 mm ID, 0.33 μm FT, Agilent Technology, Germany) with a temperature program starting from 40 to 300°C, with a heating rate of 4°C min^−1^ and the maximum temperature held for 45 min. The carrier gas was Helium with a flow rate of 1.0 mL min^−1^. All samples were measured in triplicates with a usual standard deviation of ≤ 0.5%0. The quality of the results was checked by measuring *n*-alkane standards (*n*-C_15_, *n*-C_20_, and *n*-C_25_) with known isotopic composition (Campro Scientific, Germany). Isotopic compositions are given in the delta notation relative to the Vienna Pee Dee Belemnite (VPDB) standard.

### DNA Extraction and Purification

Due to the extremely low amount of biomass in the core samples, 10 g of powdered sample material was used to extract the total genomic DNA with the DNeasy® PowerMax® Soil Kit (QIAGEN, Venlo, Netherlands). Afterwards, the obtained DNA was dissolved in 5 mL DNA-free water (Carl Roth, Karlsruhe, Germany). For each sampling depth, three independent samples were taken from different positions of the core horizon as technical triplicate. The 5 mL DNA solution was concentrated to 100 μL by an Eppendorf Concentrator Plus (Eppendorf AG, Hamburg, Germany). The Genomic DNA Clean & Concentrator™-10 (Zymo Research, Irvine, CA) was utilized to remove humic acids and other substances that may inhibit the polymerase chain reaction (PCR). Two DNA extractions were done from separated sample duplicates. DNA from 1 mL DNA-free water (Carl Roth, Karlsruhe, Germany) was extracted as a negative control using the same DNA extraction approach.

In addition to the core material, ~1 L of the fluid samples from the pump test were filtered (0.2 μm) to collect insoluble particles. The total genomic DNA trapped on the filters was extracted by the FastDNA™ SPIN Kit for Soil and the FastPrep® Instrument (MP Biomedicals, Santa Ana, CA) with standard protocols. The FastPrep® Instrument homogenizing time and the homogenizing speed were modified to 30 s and 5.5 m s^−1^ according to Liu et al. (2018).

### Quantitative PCR

Total microbial abundance was estimated by determining the number of bacterial 16S rRNA gene copies using quantitative polymerase chain reaction (qPCR) targeting the V3 region of the 16S gene with the primer pairs 341F (5′-CCTACGGGAGGCAGCAG−3′) and 534R (5′-ATTACCGCGGCTGCTGG-3′) (Degelmann et al., 2010). The qPCR Master Mix consisted of 10 μL SYBR® FAST qPCR Master Mix (2X) Universal (KAPA Biosystems, Wilmington, Massachusetts, USA), 5.92 μL PCR water, 0.04 μL forward primer (100 μM), 0.04 μL reverse primer (100 μM), and 4 μL template. The qPCR was programmed as 3 min at 95°C, 40 cycles of 3 s at 95°C, 20 s at 60°C, 30 s at 72°C, and 3 s at 80°C for the plate read. A cloned 16S rRNA gene fragment from *Escherichia coli* was used as standard. The qPCR was conducted on a CFX96 real-time thermal cycler (Bio-Rad Laboratories Inc., USA) and the analysis of the quantification data was performed with the CFX Manager™ software (Bio-Rad Laboratories Inc., USA). The concentration range of the standard was optimized and set from 10^3^ to 10^7^ 16S rRNA gene copies. The *R*^2^-value of the standard curve line was 0.994–0.997.

### Illumina MiSeq Amplicon Sequencing

The 16S rRNA gene was amplified with OptiTaq™ polymerase (Roboklon, Berlin, Germany) which has a proofreading capability due to the extremely low concentration of extracted total genomic DNA. The PCR reaction solution consisted of 2.5 μL 10x Buffer Pol C, 0.125 μL OptiTaq™ polymerase, 1 μL dNTP Mix (5 mM each), 1 μL MgCl_2_ (25 mM), 17.075 μL PCR water, 0.3 μL bovine serum albumin, 0.25 μL forward primer (20 μM), 0.25 μL reverse primer (20 μM) and 2.5 μL template. Unique combinations of barcode-tagged 515F (5′-GTGCCAGCMGCCGCGGTAA-3′) and 806R (5′-GGACTACHVGGGTWTCTAAT-3′) (Caporaso et al., 2011) primers were assigned to each sample. PCR amplifications were performed in volumes of 25 μL on a T100™ thermal cycler (Bio-Rad Laboratories Inc., USA) under the following conditions: 5 min at 95°C, 35 cycles of 30 s at 95°C, 45 s at 56°C, 60 s at 72°C, and a final extension step of 7 min at 72°C. A cloned 16S rRNA gene fragment from *E. coli* was used as positive control. Non-template controls were included with each PCR run. The PCR products were cleaned up with AMPure XP magnetic beads (Beckman Coulter GmbH, Krefeld, Germany). After measuring the DNA concentration with a CLARIO star® plate reader (BMG LABTECH GmbH, Ortenberg, Germany) PCR products were pooled in equimolar amounts. The pooled DNA solution was concentrated with Eppendorf Concentrator plus (Eppendorf AG, Hamburg, Germany) to meet the requirement of the Illumina MiSeq high-throughput sequencing. The final pooled DNA concentration was 77.05 ng μL^−1^.

### Bioinformatics and Statistical Analysis

Sequencing was performed by Eurofins Scientific SE (Luxembourg) on an Illumina MiSeq (2 × 250 bp). Dual-indexed reads were demultiplexed using CutAdapt (Martin, 2011) allowing for 10% errors in the primer and no errors in the barcodes. Individual samples were processed according to the DADA2 pipeline (Callahan et al., 2016). This includes an initial sequence truncation (250 bp forward reads; 200 bp reverse reads). The quality-filtered reads were used to generate an error model that was applied for dereplication, sample inference, and merging of the paired-end reads. All final sequences had a standardized read-orientation and a minimum length of 200 bp. The sequence table was created and potential chimera were filtered using a *de novo* approach. The resulting amplicon sequence variants (ASVs) were imported into the QIIME2 framework (Bolyen et al., 2019) which facilitated the SILVA taxonomy database (v132) (Quast et al., 2013) and VSEARCH (Rognes et al., 2016) to assign taxonomic units. Singletons and OTUs assigned to chloroplasts and mitochondria were removed from the obtained OTU table. After the filtering processes, 11,063,679 sequences were obtained in the 16S rRNA gene library in total. The read numbers ranged between 12,999 and 243,092 with a mean value of 99,985. The resulting ASV table was manually scanned for potential contaminant taxa based on the negative control, resulting in the removal of ASVs belonging to the taxa *Escherichia, Undibacterium, Methylophilaceae, Comamonadaceae, Ralstonia*, and *Novosphingobium*. Due to the low biomass environment and the high susceptibility of contamination introduction via the Power Soil Max kit (Sheik et al., 2018), comparison of drill core and pump test data was used as an additional screening approach and resulted in the removal of additional contamination ASVs (including *Lawsonella* and *Staphylococcus*). The final ASV table was rarified to a sequencing depth of 5,541 sequences (lowest available sequencing count) for alpha diversity estimation. Microbial diversity in each sample was assessed by calculating Shannon H and Shannon EH indices using the phyloseq package in R (McMurdie and Holmes, 2013). Beta diversity was determined by the non-metric multidimensional scaling (NMDS) using Bray Curtis distances with PAST3 (Hammer et al., 2001). Sequencing data was deposited at the European Nucleotide Archive (http://www.ebi.ac.uk/ena) under the accession numbers PRJEB22478 (ERS4382097 to ERS4382146 and ERS4382395 to ERS4382400).

### Correlation and PCA of Microbial Genera and Lipid Biomarkers

The correlation and Principal Component Analysis (PCA) were performed using the software PAST3 (Hammer et al., 2001). Statistical examinations considered samples investigated both DNA and lipid biomarkers. More specifically, correlations between TOC, TIC divided into TIC-Dolomite restricted to the Cypris Fm. and TIC-Siderite restricted to the Main Seam Fm., TN, the TOC/TN-ratio, all microbial genera with a relative abundance >5%, detected functional genera (<5%) related to the methane-, sulfur- and iron-cycles and all detected lipid biomarkers were investigated. The resulting correlation coefficients (*r*) are based on linear regression (Pearson) and statistical significance was reported as a *p*-value. Correlations with a *p* > 0.05 were considered to be non-significant. The PCA was carried out for TOC, TIC-Dolomite, TIC-Siderite, TN, TOC/TN-ratio, all genera >5%, additional detected functional genera (<5%) and all predominating or relatively constantly distributed lipid biomarkers. The parameters were normalized to 1 with the PAST3 function “row normalized length” and the PCA calculated on the correlation matrix. Compounds not detected were treated as zero.

## Results

### Stratigraphy and Sample Material

**Figure 2:**
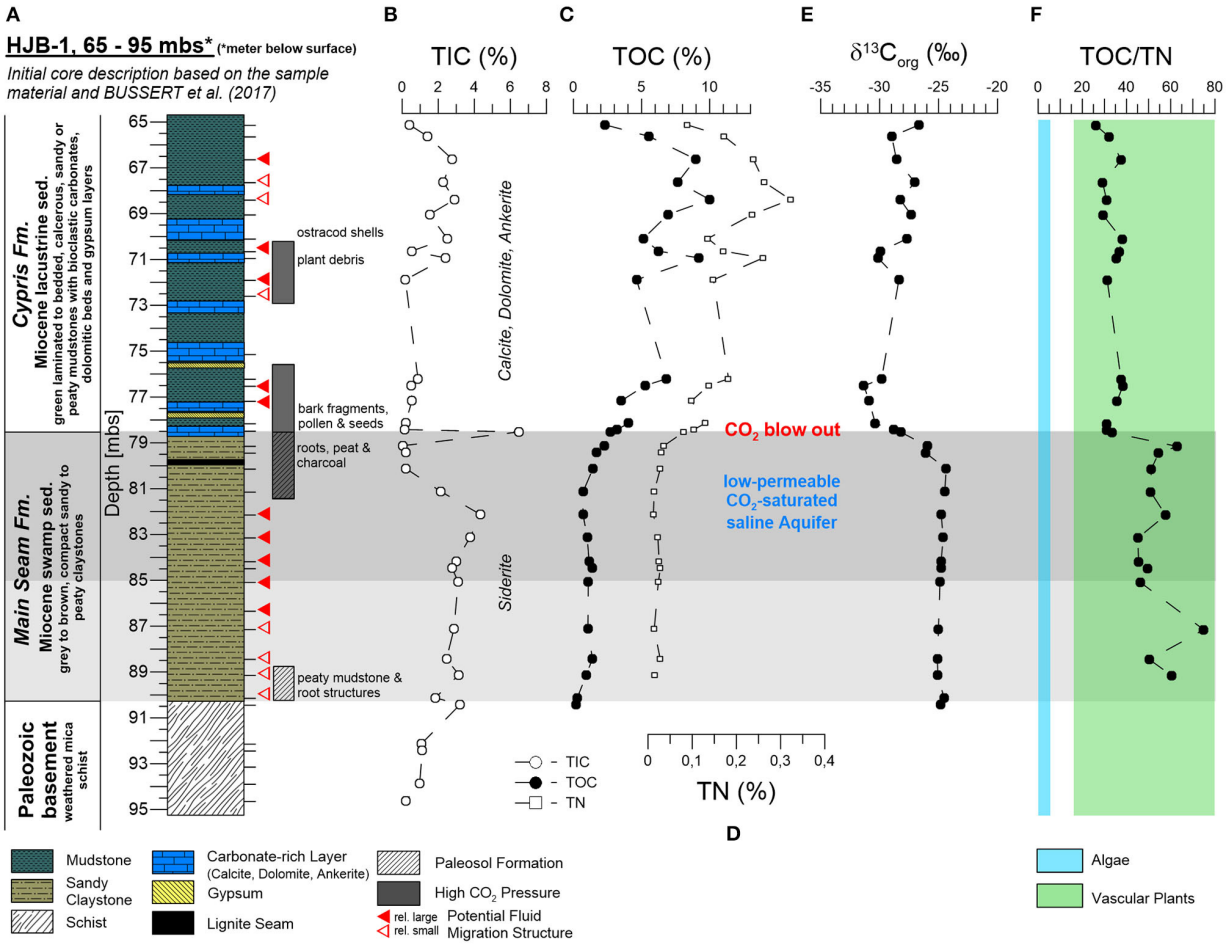
Investigated core section of the Hartoušov mofette core HJB-1 (2016) between 65 and 95 mbs with depth profiles of bulk sedimentological parameters. **(A)** Stratigraphical and lithological description based on visual inspection of the sample material and on data from Bussert et al. (2017), **(B)** total inorganic carbon (TIC), **(C)** total organic carbon (TOC), **(D)** total nitrogen (TN), **(E)** bulk δ^13^C_org_, and **(F)** the TOC/TN ratio.

The core section between 65 and 95 mbs lithologically consisted of three different units which were from the bottom to the top: (i) a weathered Paleozoic mica schist (95–91.5 mbs, Paleozoic basement), (ii) sandy to peaty Early Miocene mudstones of the Main Seam Formation (Fm.) with lignite fragments and root structures suggesting paleosol horizons in the lower and upper sections (91.5–78.5 mbs), and (iii) laminated, calcareous, sandy or peaty Early Miocene mudstones interbedded with bioclastic carbonates, dolomite to ankerite beds, and gypsum layers of lacustrine origin (78.5–65 mbs) belonging to the Cypris Fm. (Bussert et al., 2017; Figure 2A). Identified macrofossils in the Cypris Fm. were bark fragments, seeds, plant debris, and ostracod shells (*Cypris angusta Rss*.) (Figure 2A and Supplementary Figure 1). Sediments from the Main Seam and Cypris Fm. revealed vein-like structures indicating potential CO_2_ ascending pathways with associated mineral alteration and precipitation. These features were siderite-rich veins and bubble structures in the Main Seam Fm. and small fractures, dykes and sills with sediment color changes in the Cypris Fm. The CO_2_ blow out during the drilling campaign occurred at the transition from the Cypris Fm. to the underlying Main Seam Fm. This indicates that at this transition a dolomite-rich layer (about 30 cm thick) that is widely distributed in the Cheb basin (Smejkal, 1984; Pešek, 2014) or the lacustrine sediments themselves act as a sealing layer for the low-permeable CO_2_-saturated saline aquifer in the upper Main Seam Fm., resulting in a zone characterized by high CO_2_ pore pressure (Figure 2A).

### Bulk Carbon and Nitrogen

Carbonates were detected in all three lithological units and are expressed in total inorganic carbon (TIC) (Figure 2B). In the Paleozoic basement and in the Main Seam Fm. the carbonates were mainly represented by zoned siderite spheres and veins that could have been precipitated from the low-permeable CO_2_-saturated saline aquifer. At the transition from the Main Seam Fm. to the Cypris Fm. carbonates were essentially absent, except of the thick (30 cm) dolomite-rich layer at 78.5 mbs. In the Cypris Fm., calcite, dolomite, and ankerite predominated. Their occurrence together with evaporitic layers indicates a lacustrine origin (Smejkal, 1984; Pešek, 2014). However, a possible early diagenetic alteration cannot be excluded.

Organic matter was not detected in the Paleozoic basement. The TOC contents of the Main Seam Fm. ranged between 0.2 and 2.3%. After a small increase the TOC contents remained relatively constant at ca. one percent before increasing to 2.3% at the top of the Main Seam Fm. (Figure 2C). In the overlying lacustrine Cypris Fm. the TOC contents were significantly higher and show strong fluctuations between 2.3 and 10%. Bulk δ^13^C_org_ data also changed with the lithological transition from the Main Seam to the Cypris Fm. showing relative constant values around −24%0 in most parts of the Main Seam Fm. and a strong decrease down to −30%0 at the top (Figure 2E). In the Cypris Fm. the organic carbon isotope signals fluctuate between −31 and −27%0.

The total nitrogen content (TN, Figure 2D) was mainly positively correlated with the TOC content (*r* = 0.99) (Supplementary Table 1). Values were low in the Main Seam Fm. ranging between 0.01 and 0.04% and increase at the top. In the Cypris Fm. TN values were significantly higher ranging between 0.08 and 0.32%. The TOC/TN ratio ranged between 45 and 75 in the Main Seam Fm. and between 26 and 41 in the Cypris Fm. (Figure 2F).

### Microbial Lipid Biomarker Signals

**Figure 3:**
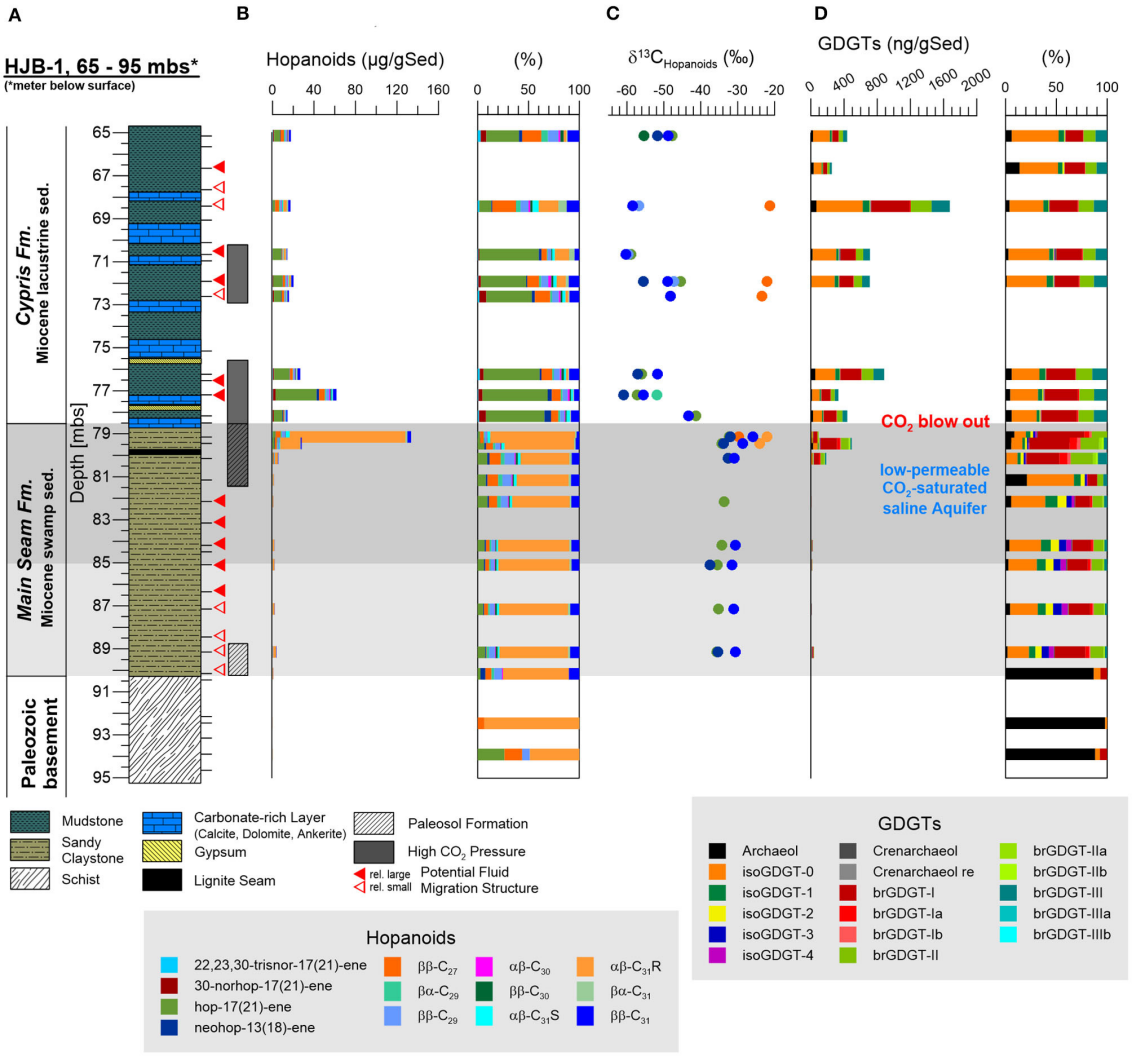
Investigated core section of the Hartoušov mofette core HJB-1 (2016) between 65 and 95 mbs with depth profiles of investigated lipid biomarkers. **(A)** Stratigraphical and lithological description, **(B)** the hopanoid distribution and its relative abundance (%), **(C)** δ^13^C_hopanoids_ of different hopanoids, and **(D)** the (core) GDGT distribution and its relative abundance.

Hopanoids, representing stabilizing and ordering components in bacterial cell membranes (Sáenz et al., 2015), could be detected as unsaturated hopenes [22,23,30-trisnor-17(21)-ene, 30-norhop-17(21)-ene, hop-17(21)-ene, neohop-13(18)-ene] and saturated hopanes with the steric 17β(H), 21β(H)-, 17β(H), 21α(H)-, and 17α(H)β(H)-configuration (ββ–C_27_, ββ–C_29_, βα–C_29_, ββ–C_30_, αβ–C_30_, ββ–C_31_, βα–C_31_, αβ–C_31_R, αβ–C_31_S) (Figure 3B). In the Paleozoic basement hopanoids were essentially absent. In the Main Seam Fm. the hopanoid signal was usually low (<6 μg gSed^−1^) and dominated by the αβ-C_31_R-hopane. However, at the top to the Main Seam Fm. the hopanoid concentrations especially the αβ-C_31_R signal distinctly increased to the highest values in the entire investigated core interval (134 μg gSed^−1^) (Figure 3B). In the Cypris Fm., hopanoids were generally more abundant (14–62 μg gSed^−1^) than in the Main Seam Fm. (excepting the top of the Main Seam Fm.) and hop-17(21)-ene supplemented by ββ-hopanes were the predominating hopanoid compounds (Figure 3B). The δ^13^C_hopanoid_ values in the Main Seam Fm. plotted in a range between −22 and −37%0 and shifted to significantly lower values between −42 and −60%0 in the Cypris Fm. (Figure 3C).

In general, GDGTs, also referred to as core GDGTs, are degradation products of membrane-spanning intact microbial lipids (IPL-GDGTs), which contain the GDGT core unit linked to one or two head groups. In contrast to the intact polar lipids (IPL-GDGTs) the GDGT core lipids are stable over geological times. Structural differences lead to a subdivision into branched GDGTs (brGDGTs) and isoprenoid GDGTs (isoGDGTs) (Schouten et al., 2013). The brGDGTs are known to originate from bacteria in terrestrial environments (soils, peats, rivers, and lakes) (Weijers et al., 2007, 2010; Tierney et al., 2012; Dirghangi et al., 2013; DeJonge et al., 2014; Freymond et al., 2017; Huguet et al., 2017; Naafs et al., 2017) and the isoGDGTs are from archaea within aquatic (marine and lacustrine) and terrestrial systems (Pancost et al., 2011; Tierney et al., 2012; Dirghangi et al., 2013; Bischoff et al., 2014; Naeher et al., 2014; Schubotz et al., 2018; Bale et al., 2019; Evans et al., 2019). Both GDGT types occurred across the whole lithological profile with isoGDGT-0, isoGDGT-1, brGDGT-I, brGDGT-II, and brGDGT-III being the most prominent (Figure 3D). In addition to the GDGTs, the archaeal membrane degradation product archaeol was also present in notable amounts. Except isoGDGT-3, all detected GDGTs showed a positive correlation with TOC (Supplementary Table 1). In the Paleozoic basement and lower part of the Main Seam Fm., GDGTs were either absent or identified at low concentrations (1–20 ng gSed^−1^). The top of the Main Seam Fm. revealed a clear predominance of brGDGTs with a dominating brGDGT-I (~200 ng gSed^−1^) and subordinated brGDGT-Ia (~37 ng gSed^−1^), brGDGT-II (~90 ng gSed^−1^), and isoGDGT-0 (~55 ng gSed^−1^). Archaeol occurs in lower amounts (~28 ng gSed^−1^). In the Cypris Fm., isoGDGT-0 ranging between 91 and 559 ng gSed^−1^ became the most predominant GDGT and brGDGT-I was found to be the second most abundant GDGT (50–462 ng gSed^−1^). Slightly increased concentrations were also detected for brGDGT-III (26–219 ng gSed^−1^) and isoGDGT-1 (11–82 ng gSed^−1^). Interestingly, brGDGT-II (28–252 ng gSed^−1^) and brGDGT-III appeared in similar amounts from this point to the top of the Cypris Fm. (78–65 mbs). Archaeol contents were low and could be positively correlated to the TOC (8–67 ng gSed^−1^; *r* = 0.88). Crenarchaeol appeared across the whole profile but in very low amounts ranging between 0 and 10 ng gSed^−1^ (Figure 3D).

**Figure 4:**
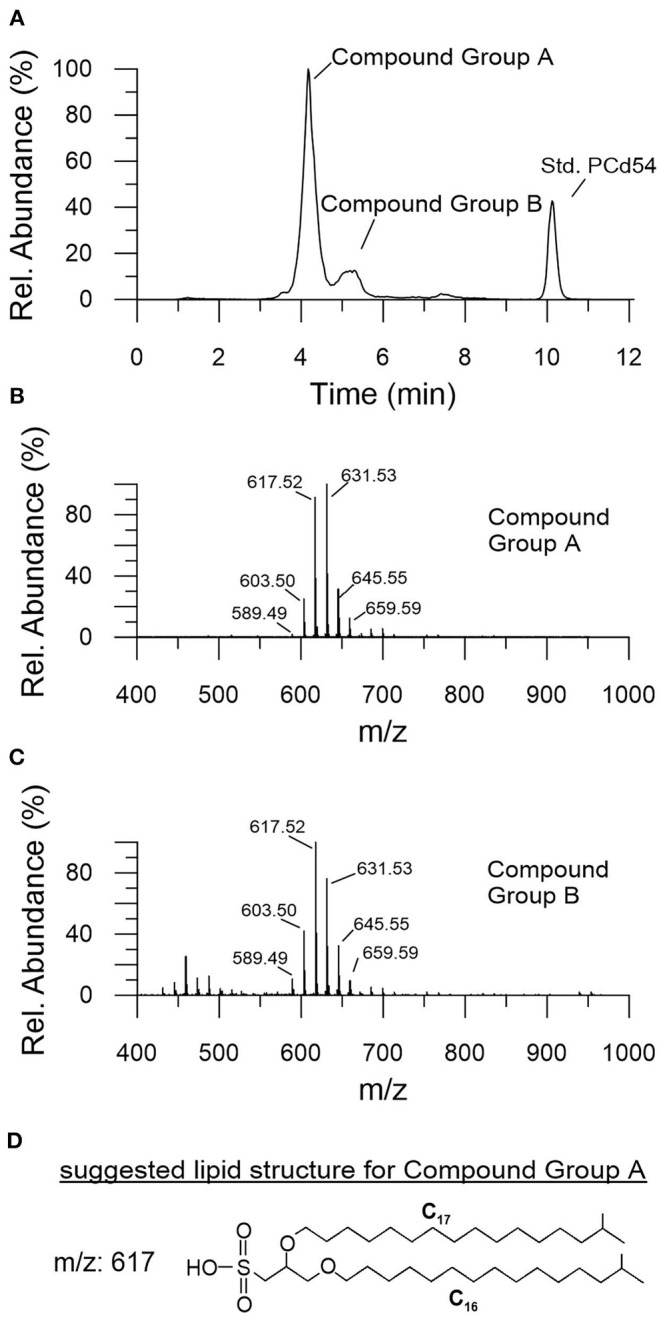
Description of the yet unknown groups of intact polar lipids (IPLs) referred to as compound group A and B. **(A)** HPLC chromatogram, **(B,C)** associated mass spectra, both showing a cluster of six individual mass peaks differing by 14 mass units with a maximum at m/z 631 or 617, respectively. **(D)** Suggested lipid structure for the mass peak at m/z 617 of compound A.

**Figure 5:**
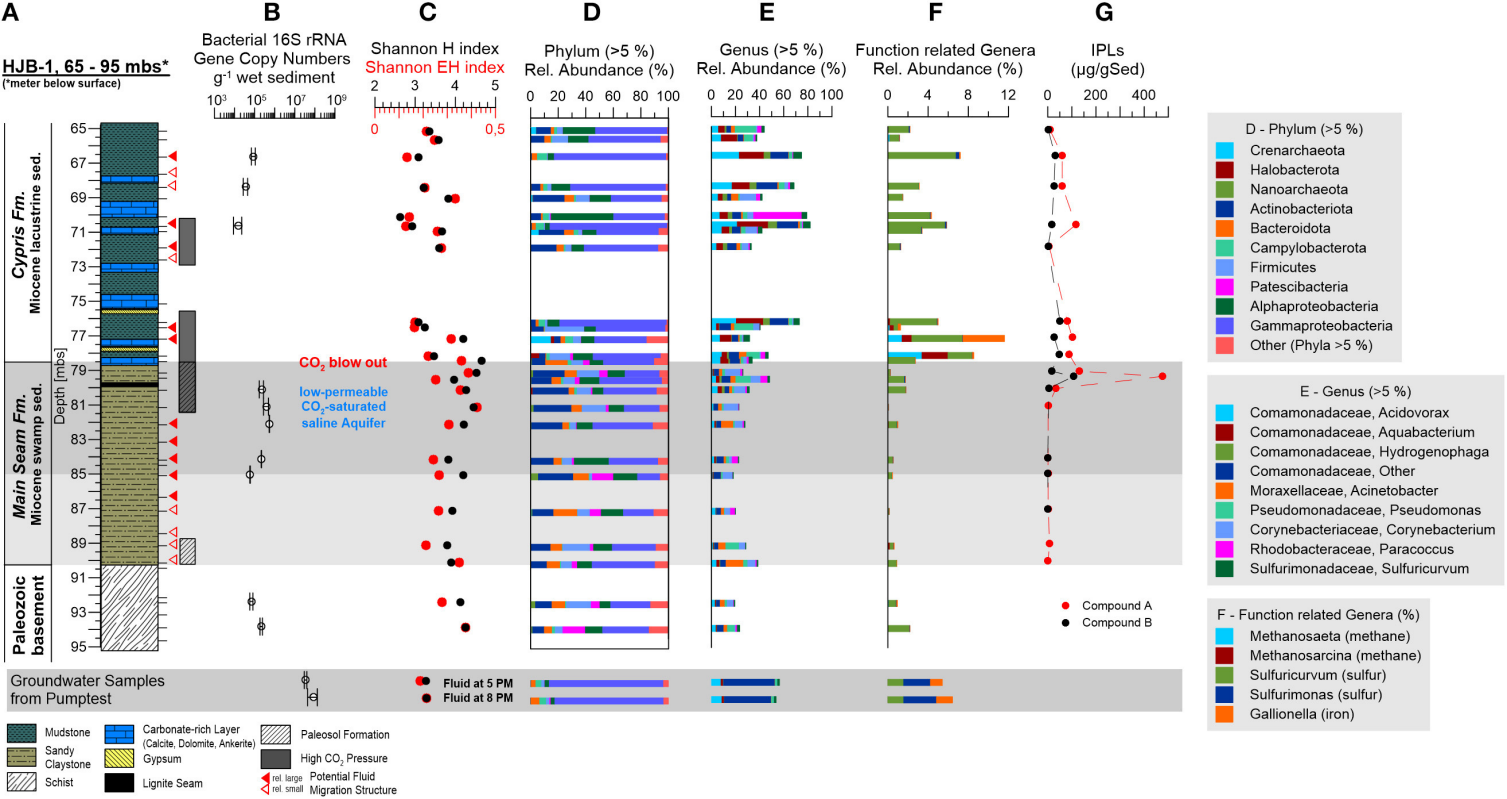
Investigated core section of the Hartoušov mofette core HJB-1 (2016) between 65 and 95 mbs with depth profiles of investigated geomicrobiological parameters and intact lipid biomarkers. **(A)** Stratigraphical and lithological description of the Hartoušov mofette core HJB-1 (2016), **(B)** bacterial 16S rRNA gene copy numbers, **(C)** the Shannon H and Shannon EH indices, **(D)** the community structure on phylum (>10%) and **(E)** genus (>5%) levels, **(F)** specific genera with respect to their metabolism, and **(G)** intact polar lipids (IPLs).

In contrast to the hopanoids and GDGTs, both commonly interpreted to represent necrotic microbial biomass, intact polar lipids (IPLs) provide information on present microbial life, since these biomarkers degrade relatively rapid after cell death (White et al., 1979; Zink et al., 2003). The chromatogram of IPLs revealed no common phospholipids, but a double peak which represented, to the best of our knowledge, unknown lipid compound groups. These groups were tentatively referred to as compound group A and B (Figure 4A). The mass spectra of these two compound groups showed a cluster of six individual mass peaks with a maximum at m/z 631 or 617, respectively (Figures 4B,C). The masses differed by 14 mass units indicating an increase of the lipid side chain length by a CH_2_-group. The individual masses of the two compound groups were essentially the same, indicating the same elemental composition. This and the close vicinity of the chromatographic signals suggested that these compounds bear the same head group but show different configurations in their side chains (e.g., OH-group vs. ether-group), causing slightly different elution behaviors. Microbial membrane lipids usually consist of a polar head group and two long-chain ether or ester side chains linked to a glycerol backbone (Mangelsdorf et al., 2019). To elucidate whether ester bond fatty acids formed the lipid side chains, MS-MS and saponification experiments [polar lipid fatty acid (PLFA) analysis] were conducted indicating that the side chains are not ester bound fatty acids and that alkyl and ether bound side chains are more likely. High resolution analysis using an Orbitrap MS provided insights on the elemental formular of the unknown compounds. This approach, together with the isotope patterns of the individual molecular masses, indicated the presence of sulfur (^32^S and ^34^S-isotope) presumably a sulfonic acid (R-SO_2_-OH) as the head group part. An example for the suggested lipid structure for the mass peak at 617 m/z within compound group A is shown in Figure 4D and further experiments have to be conducted to determine the full structure of compound groups A and B. Both lipid groups were almost absent in the basement and Main Seam Fm. (0–7.6 μg/gSed; Figure 5G). However, they were most abundant (475.2 μg/gSed) at the top of the Main Seam Fm. with its high CO_2_ pressure. In the Cypris Fm. the signal decreased significantly again but was still a bit higher in CO_2_ influenced core intervals (up to 116 μg/gSed; Figures 5A,G).

### Abundance of Microorganisms

Total microbial abundance was assessed by measuring the number of bacterial 16S rRNA gene copies per g^−1^ wet sediment across the evaluated core section. Microbial abundance ranged between of 10^4^ and 10^5^ 16S rRNA gene copies g^−1^ wet sediment in the Paleozoic basement (93.9 and 92.4 mbs), the low-permeable CO_2_-saturated saline aquifer in the upper Main Seam Fm. (85.1, 84.2, 82.2, 81.1, and 80.2 mbs) and in the upper part of the investigated Cypris Fm. showing fluid migration structures (70.7, 68.4, and 66.7 mbs) (Figures 5A,B). No data was obtained for the other samples due to low biomass or the presence of inhibitors. The fluid filter samples from the pump test contained gene copy numbers in a range of 10^7^ 16S rRNA gene copies L^−1^ (Figure 5B).

### Microbial Community Composition

Alpha diversity of the microbial community for the whole lithological profile was assessed by calculating the Shannon H and evenness indices (diversity) per 5,541 sequences. The Shannon H index ranged between 2.6 and 4.7 and the Shannon EH index (evenness) ranged between 0.1 and 0.4 (Figure 5C). Shannon H and Shannon EH indices were found to be higher in the Paleozoic basement, the Main Seam Fm. and at the bottom of the Cypris Fm. (3.8–4.7 and 0.2–0.4) and lower in most of the upper investigated Cypris Fm. (2.6–3.6 and 0.1–0.2) (Figure 5C). Shannon H and EH indices for the fluid filter samples ranged around 3.3 and 0.2 (Figure 5C).

Microbial communities across the analyzed core section were found to be dominated by Bacteria (78–99%) (Figure 5D). The bacterial community in the Paleozoic basement and Main Seam Fm. was characterized by large abundances of *Proteobacteria*, specifically *Gammaproteobacteria* (16–45%) and *Alphaproteobacteria* (8–25%). In addition, *Actinobacteria* (8–27%), *Bacteroidota* (4–17%), and *Firmicutes* (2–20%) were found to be enriched in these layers. *Gammaproteobacteria* (37–85%) and *Alphaproteobacteria* (3–45%) were even more abundant in the Cypris Fm., while the detection of *Actinobacteriota, Bacteroidota*, and *Firmicutes* varied depending on the evaluated core section. Core sections, where these taxa were identified at abundances similar to the Main Seam Fm., will be in the following text referred to as major Cypris Fm. (65.2, 65.7, 69.1, 71.0, 71.9, and 76.5 mbs), while core sections where *Actinobacteriota, Bacteroidota*, and *Firmicutes* were discovered at low relative abundances (66.7, 68.4, 70.7, and 76.2 mbs) will be referred to as intercalated zones.

Archaeal abundances were generally low (0–3%), but appeared to be elevated at a depth of 65.2 mbs (4%), 71.0 mbs (6%), 77.2 mbs (18%), 78.2 mbs (6%), and 85.1 mbs (6%). Archaeal communities were dominated by the phyla *Crenarchaeota* (average 1.3%, up to 14.8%) and *Halobacterota* (up to 6.0%). In addition, *Nanoarchaeota* (3.0–5.5%) were enriched in two deeper core sections (85.1 and 92.5 mbs) (Figure 5D).

The community of the groundwater filter samples from the pump test was dominated by *Gammaproteobacteria* (81%) with low amounts of *Bacteroidota* (5%) and *Campylobacterota* (5%) and showed similarities to the community structure of the intercalated zones in the Cypris Fm. (Figure 5D).

The microbial community was evaluated in more detail by taking a closer look at the genera with a relative abundance of more than 5%. Members belonging to the family *Comamonadaceae* known from soil and aquatic environments represented the largest fraction of Proteobacteria across the entire analyzed core. This group includes the facultative anaerobic and potentially iron-oxidizing taxon *Acidovorax*, the biofilm-forming *Aquabacterium*, the putative facultative autotrophic genus *Hydrogenophaga*, and several uncharacterized *Comamonadaceae* genera (Figure 5E). Other genera identified at relative abundance of >5% were *Acinetobacter, Pseudomonas, Corynebacterium, Paracoccus*, and *Sulfuricurvum* (Figure 5E).

In the Paleozoic basement and Main Seam Fm. most dominant genera (>5%) did not exceed 50% of the total relative abundance and were represented by *Acinetobacter* (0.2–15%), *Pseudomonas* (0.6–13%), *Corynebacterium* (1–13%), unknown *Comamonadaceae* (3–7%), *Paracoccus* (0.1–6%), and *Acidovorax* (1–5%). The taxa *Acidovorax, Aquabacterium, Hydrogenophaga, Pseudomonas*, and *Sulfuricurvum* were enriched at the transition from the Main Seam to the Cypris Fm. (Figure 5E). The major Cypris Fm. was characterized by the occurrence of genera also found in the Paleozoic basement and Main Seam Fm. [i.e., *Acinetobacter* (0.5–6%), *Pseudomonas* (1–17%), *Corynebacterium* (1–14%), unknown *Comamonadaceae* genera (3–10%), *Paracoccus* (0.1–40%), and *Acidovorax* (4–9%)]. However, this was supplemented by an increased occurrence of *Aquabacterium* (2–13%), *Hydrogenophaga* (0.4–4%), and *Sulfuricurvum* (0.4–5%). The intercalated zones were dominated by *Aquabacterium* (15–25%), *Acidovorax* (17–23%), unknown *Comamonadaceae* (15–18%), *Hydrogenophaga* (5–8%), and *Sulfuricurvum* (3–7%) whereas the other genera were significantly decreased. The groundwater filter samples were dominated by unknown *Comamonadaceae* genera (41%) and contained low amounts of *Acidovorax* (8%), *Aquabacterium* (2%), *Pseudomonas* (2%), and *Sulfuricurvum* (2%) (Figure 5E).

Due to the paleo-environmental conditions of the investigated lithological profile, the hydro-geochemical composition of the low-permeable CO_2_-saturated saline aquifer with high CO_2_, ${\text{SO}}_{4}^{2-}$, and Fe^2−^ concentrations (Bussert et al., 2017), and the previous microbiological surface investigations from the Hartoušov and Bublák mofette systems (Beulig et al., 2015; Krauze et al., 2017; Liu et al., 2018), the presence of microorganisms involved in methane-, sulfur-, and iron- cycling were expected. However, only small fractions belonging to these microbial groups could be detected. Methanogens were represented by *Methanosarcina* (0–3%) and *Methanosaeta* (0–3%) and were mainly detected at the base of the Cypris Fm. (Figure 5F). In spite of their comparably low abundance, sulfur-cycling genera formed the most abundant group of functional taxa and consisted of the sulfur-oxidizing genera *Sulfuricurvum* and *Sulfurimonas*. *Sulfuricurvum* was frequently detected across the evaluated core sections (0.4–8%) and was especially abundant at the transition from the Main Seam to the Cypris Fm. and in other intervals of the Cypris Fm. (10%). *Sulfurimonas* was most abundant in the groundwater filter samples (3%) (Figure 5F). A notable iron-cycling genus was the putative iron-oxidizer *Gallionella*. While *Gallionella* abundances were generally low (<0.3%), sequences belonging to this genus were frequently detected at the base of the Cypris Fm. (4%) and in the groundwater filter samples (1%) (Figure 5F).

**Figure 6:**
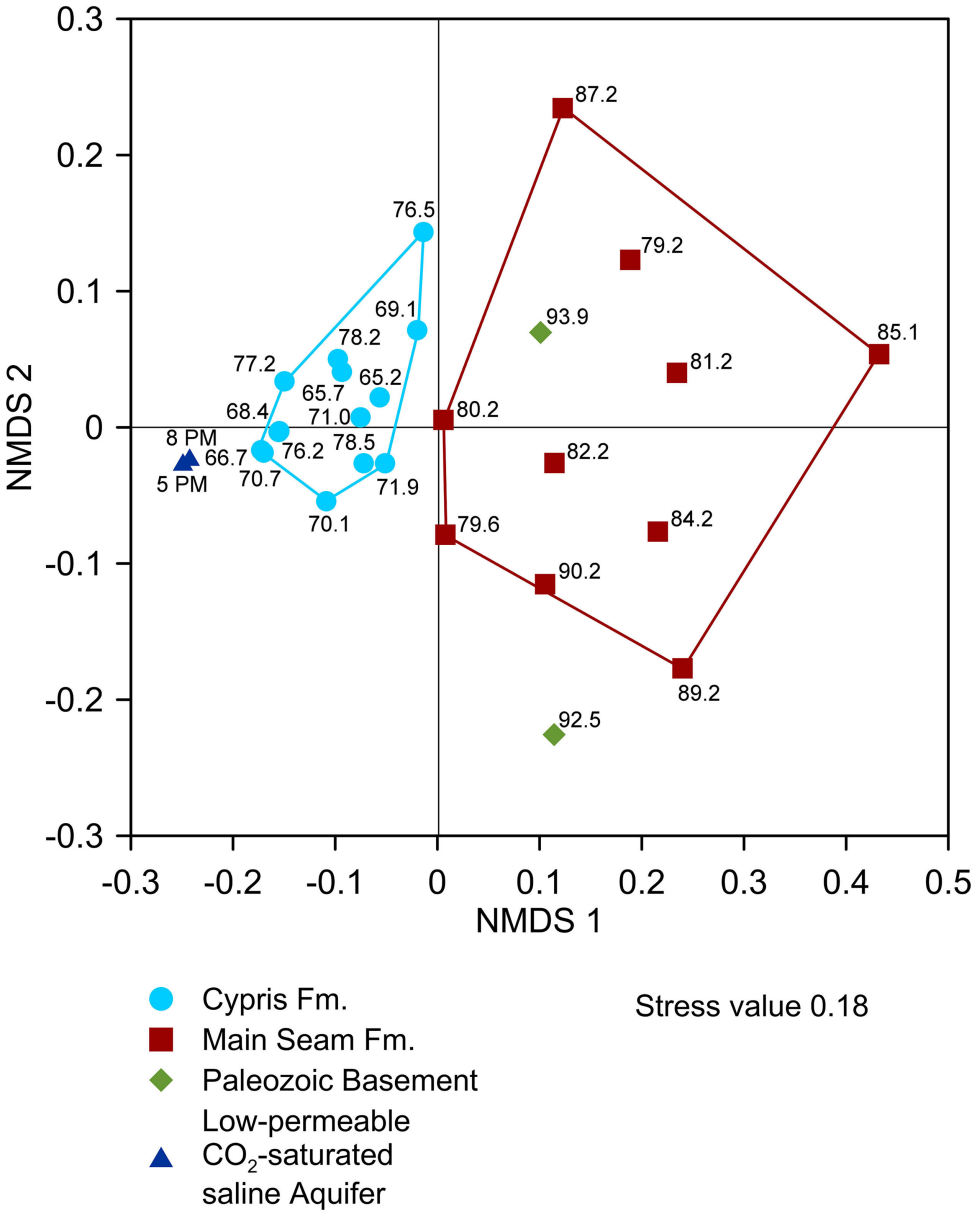
Beta diversity obtained by non-metric multidimensional scaling (NMDS) based on the Bray-Curtis dissimilarity to compare the relative abundance of microorganisms within the samples with respect to their distribution within the lithological profile, i.e., Paleozoic basement, Main Seam Fm., and lacustrine Cypris Fm. as well as the groundwater of the deep CO_2_-saturated aquifer.

Comparison of the microbial distribution across the lithological profile using non-metric multidimensional scaling (NMDS) based on the Bray-Curtis (stress 0.18) identified two major clusters associated with the Main Seam and Cypris Fm. (Figure 6). Samples from the Paleozoic basement clustered closer to the Main Seam Fm. and the groundwater filter samples clustered closer to the intercalated zones of the Cypris Fm. (Figure 6).

### Statistical Analysis of Environmental Parameters, Lipid Biomarkers, and the Microbial Community Structure

Correlation analysis of the bulk elemental parameters, the most abundant microbial genera (>5%), detected functional genera (<5%) and all identified lipid biomarkers revealed various positive correlations (Supplementary Table 1). Within the environmental parameters TIC-Dolomite was found to correlate with TOC and TN. These environmental parameters were found to be positively correlated to the occurrence and relative abundance of the taxa *Comamonadacceae, Acidovorax, Aquabacterium, Hydrogenophaga*, and *Sulfuricurvum*, all predominating GDGTs (isoGDGT-0, isoGDGT-1, brGDGT-I, brGDGT-II, and brGDGT-III), most of the rarely detected isoGDGTs (isoGDGT-2, isoGDGT-4, Crenarchaeol, and Crenarchaeol region-isomer) and archaeol (Supplementary Table 1). Most importantly the above mentioned taxa and lipids were also positively correlated with each other (Supplementary Table 1). Since, the correlated genera belong to the domain of bacteria and isoGDGTs are known to be produced by archaea (Schouten et al., 2013) the results evidently represent a spurious correlation, meaning that the investigated lipids cannot be directly assigned to the identified microorganisms. In general, all of these parameters increase at the top of the Main Seam Fm. and exhibit all around higher abundances in the Cypris Fm. (Figures 2C,D, 3B,D, 5D–G). Hence, it is likely that the occurrence pattern of both lipid and microbial abundances is not correlated to each other, but rather driven by the TOC content.

**Figure 7:**
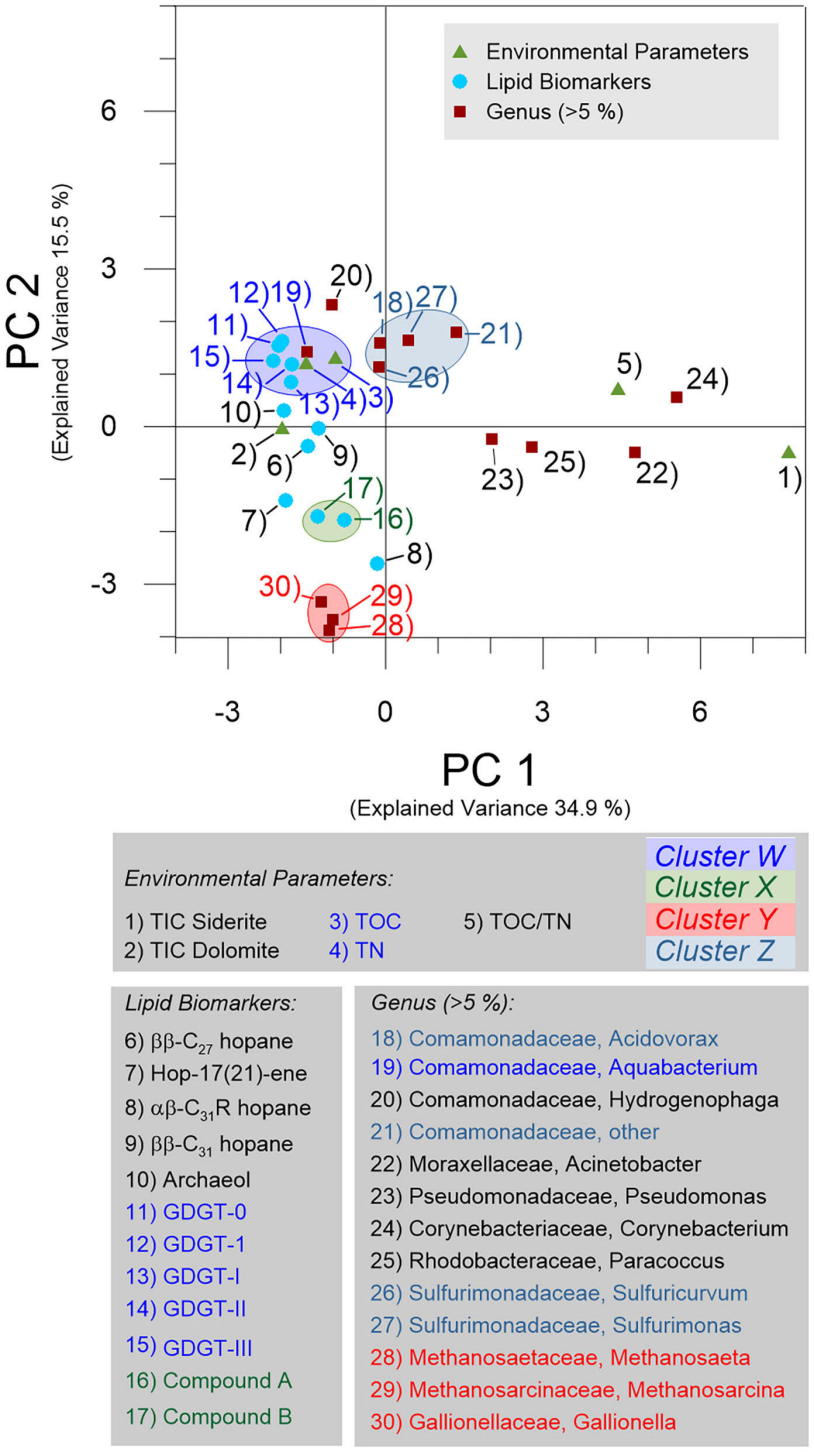
Biplot showing the principal component (PC) loadings for PC1 and PC2 obtained from principal component analysis (PCA) of the environmental parameters, selected microbial genera and predominating microbial lipid biomarkers.

The conducted principal component analysis (PCA) identified one predominating and several minor environmental factors, which may influence the distribution and relation of the identified microbial genera and lipid biomarkers (Figure 7 and Supplementary Figure 2A). In the process, the first two principal components (PCs) explained a cumulative variance of 50.4% with an outstanding explained variance of 34.9% for PC1 and 15.5% for PC2 (Figure 7). TIC-Siderite together with the taxa *Acinetobacter, Pseudomonas, Corynebacterium*, and *Paracoccus* (all of which represent the Paleozoic basement and Main Seam Fm. characterized by a low TOC content) plotted on the positive PC1 axis. In contrast, the environmental parameters TIC-Dolomite, TOC and TN (which are closely associated with the Cypris Fm.) as well as the genera *Aquabacterium, Hydogenophaga, Acidovorax, Sulfuricurvum, Methanosaeta, Methanosarcina*, and *Gallionella* and all detected lipid biomarkers plotted on or near the negative PC1 axis (Figure 7). The principal component analysis resulted into four small clusters referred to as cluster W, X, Y, and Z (Figure 7). Cluster W was represented by a negative PC1 and a positive PC2 value range and was comprised of TOC, TN, all GDGTs (isoGDGT-0, iso-GDGT-1, brGDGT-I, br-GDGT-II, and brGDGT-III) and the genus *Aquabacterium*. In addition, archaeol and the genera *Hydogenophaga* plotted close to cluster W (Figure 7). Clusters X and Y plotted both in the negative ranges of PC1 and PC2. Cluster X contained the putative unknown lipid biomarker compound groups A and B (Figure 7) and Cluster Y included the genera *Methanosaeta, Methanosarcina*, and *Gallionella* (Figure 7). All parameters related to these two clusters were abundant at the top of the Main Seam or at the bottom of the Cypris Fm. (Figures 3B,D, 5F). Cluster Z was located near cluster W and comprised of frequently identified taxa (both in the core and the groundwater), namely the unknown genera of the family *Comamonadaceae, Acidovorax, Sulfurimonas*, and *Sulfuricurvum*, but did not include any environmental parameters or lipid biomarkers (Figure 7).

The PCA clustering suggests that the parameters, taxa, and lipids aggregating in and around clusters W and Z are positively correlated to TOC. The positive correlation of these parameters with the TOC content (Figure 7 and Supplementary Table 1) supports the TOC as major environmental shaping factor represented by PC1 and becomes obvious when comparing the depth distributions of the TOC content (Figure 2C) with the PC1 loadings (Supplementary Figure 2C).

## Discussion

### Differentiation Into Present and Past Microbial Signals

Data analysis identified no meaningful correlation between the present microbial community and the lipid biomarkers but a positive correlation of both with the TOC content. This is shown by the PCA analysis plotting biomarker signals and microbial community data essentially into different clusters (Figure 7).

Nevertheless, in order to further prove that the occurrence of core GDGTs is not linked to the ascending CO_2_, we compared our GDGT results to the findings of Oppermann et al. (2010), who investigated the distribution of intact GDGTs in a surface interval of a natural CO_2_ vent in comparison to a non-influenced reference site. They found a 10 times higher amount of intact GDGTs (branched and isoprenoid) in the CO_2_ vent compared to the reference site and the vent site was dominated by brGDGT-I, -II, and -III as well as significantly increased amounts of isoGDGTs with 1, 2, 3, and 4 cyclopentyl rings relative to isoGDGT-0 (no ring) and crenarchaeol (Oppermann et al., 2010). However, a similar core GDGT distribution was not detected in the intervals of high CO_2_ pressure between 70 to 81 mbs in the Hartoušov core (Figures 3A,D). While brGDGTs are also dominated by brGDGT-I, -II, and –III, isoprenoid GDGTs are dominated by isoGDGT-0 and isoGDGT-1,−2,−3, and−4 are in general very low or absent (Figure 3D). This finding and the strong correlation of GDGTs to the TOC indicates that brGDGTs are more likely restricted to surficial environments like soils, peats, and lakes and therefore represent a past microbial signal (Pancost et al., 2011; Tierney et al., 2012; Dirghangi et al., 2013) and that the isoGDGTs are mainly of syn-sedimentary origin rather than from archaea related to the ascending mantle-derived CO_2_. In addition, the occurrence of archaea at the bottom of the Cypris Fm. (Figures 5D,F) does not result in increased amounts of archaeol and isoGDGT-0 and−1 at this interval (Figure 3D). Since these lipids are reported to represent methanogens in subaquatic (lakes) and subaerial (peats, soils, permafrost) systems (Pancost et al., 2011; Tierney et al., 2012; Dirghangi et al., 2013; Bischoff et al., 2014), the assumption of a syn-sedimentary origin for most of these isoGDGTs seem to be very likely. However, a minor production of archaeol or isoGDGT-0 by *Methonasaeta* and *Methanosarcina* at the base of the Cypris Fm. cannot be ruled out (Bischoff et al., 2014; Evans et al., 2019).

The lack of meaningful correlations between hopanoids and bacterial genera (Supplementary Table 1) and the compositional change of the dominating hopanoid types from the Main Seam Fm. (αβ–C_31_R-hopane dominated) to the Cypris Fm. [Hop-17(21)-ene dominated] (Figure 3B) suggests that the hopanoid distribution is also the result of compound preservation during time of deposition. This suggestion is confirmed by the strong shift of the δ^13^C_hopanoid_ values (Figure 3C) showing a clear decrease related to the transition from the Main Seam to the Cypris Fm. and not to the occurrence of increased CO_2_ concentrations (Figures 3A,B).

In contrast to the GDGTs and hopanoids intact polar lipids (IPLs) are usually interpreted as markers for living microorganisms (White et al., 1979). Two uncommon and putative unknown IPL groups A and B were detected in the Hartoušov core material (Figure 5G). First structural assessment suggests that they bear a sulfur-containing head group (presumably a sulfonic acid group) and ether- or alkyl-linked long hydrophobic side chains (Figure 4D). This side chain structure would explain why they were not detected during the PLFA analysis, only targeting ester-linked side chains. The fact that two clusters of up to six compounds representing individual lipids with the same head group but different side chain lengths (-CH_2_- differences) were detected, point to the origin of these lipids from bacteria or a single bacterium. Archaea also contain ether-linked side chains, but they do not show this side chain length variability (Mangelsdorf et al., 2019). Due to the ether-linked side chains with its higher stability against initial degradation, it might be argued that the potential of these IPLs to act as a life marker is restricted (Logemann et al., 2011). However, these compounds show their highest concentration in the sediments with increased CO_2_ abundance especially below the interface between the Main Seam and the Cypris Fm. (Figure 5G). This suggests that the source bacteria are related to the high CO_2_ concentrations in the deep subsurface of the Hartoušov mofette and that these uncommon lipids act here as life markers, because their intact preservation since millions of years is not very likely. It can only be speculated whether these lipids (Figure 4D) are derived from sulfur-cycling genera, which were significantly detected in the same intervals although in different amounts than the lipid markers (Figures 5F,G). Further analysis has to be conducted to elucidate the exact structure and origin of these uncommon membrane lipids.

In summary, the investigation of the microbial community structure and the microbial lipid biomarkers in the deep sediments of the Hartoušov mofette system reveals a community distribution that does not significantly correlate to the profiles of the identified lipid biomarkers (Figures 3B–D, 5D–G and Supplementary Table 1). Thus, our findings suggest that GDGTs and hopanoids are not remnants of a currently living microbial community, but represent a signal of syn-sedimentary past microbial communities. Hopanoids more likely represent necrotic remains of bacteria (Ourisson et al., 1979; Summons et al., 2006; Naeher et al., 2014; Talbot et al., 2016) and GDGTs were produced by microorganisms living in soils (Blaga et al., 2009; Weijers et al., 2009, 2010) or lacustrine systems (Blaga et al., 2009). Two groups of unknown IPLs point to the present of living microorganisms related to the ascending CO_2_. Overall, our results indicate that present microbial life in the Eger Rift subsurface is limited, as low microbial abundances (from below the detection limit to amounts between 10^4^ and 10^5^ copy numbers g^−1^ wet sediment), low microbial diversity (low Shannon H indices) and the absence of common IPLs suggest low turnover rates. Hence, the limited microbial activity is not able to overprint the past biomarker signal. Nevertheless, the recovery of microbial DNA over the whole lithological profile, the albeit limited, but persistent presence of bacterial 16S rRNA genes, the identification of a specific microbial community and the occurrence of two uncommon IPL groups support the existence of a distinct deep biosphere. An explanation for the identification of DNA, while no common IPLs could be detected, are the different detection limits regarding these two analytical approaches. The DNA amplicon sequencing approach is able to amplify low amounts of DNA and in combination with the higher than usual amount of sample material used for DNA extraction here (10 g), this results into a significant lowering of the detection limit for the DNA analysis compared to the lipid analysis. In contrast, the lipid analysis does not contain an amplification step, resulting in lower sensitivity even when increasing the sample amount for the lipid biomarker extraction to 80 g.

### Depositional Environment and Past Microbial Signatures

Based on the lithological description by Bussert et al. (2017), the Early Miocene Main Seam Fm. is characterized by terrestrial sediments deposited in a swamp environment. This is confirmed by the low TN values, the high TOC/TN ratio and the bulk δ^13^C_org_ values around −26%0 indicating that vascular C3 plants are the major source of organic matter (OM) (Meyers, 1997, 2003; Figure 2). The abundance of roots, peat, and charcoal in the sample material (Figure 2A) as well as increases in the TOC contents suggests two intervals of paleosol and peat formation at the bottom and the top of the Main Seam Fm. (Figure 2C).

The Early Miocene Cypris Fm. is described as lacustrine deposit, which is lithologically more heterogenous with phases in which carbonate precipitation interrupted the predominant siliciclastic sedimentation (Bussert et al., 2017). The high and fluctuating TOC contents point to changes in OM production related to changing environmental conditions (Figure 2C). Fossil ostracod shells and higher TN values indicate planktonic biomass (Meyers, 1997, 2003; Figures 2A,D). Compared to the Main Seam Fm., the TOC/TN ratio is indeed significantly lower, but with values between 25 and 40 still in the range of land plant material (Figure 2F). This indicates that the lacustrine OM is a mixture of autochthonous aquatic and allochthonous terrestrial biomass. The depletion in bulk δ^13^C_org_ (Figure 2E) points to an increased input and/or preservation of ^13^C-depleted OM, such as lignins, lipids, or their degradation products (Gleixner et al., 1993; Werth and Kuzyakov, 2010), the preservation of isotopic lighter freshwater plankton (Gaines et al., 2009) and/or to early diagenetic microbially induced carbon isotope fractionation, e.g., by methanogenesis, methanotrophy, or acetogenesis (Whiticar, 1999; Boetius et al., 2000; Conrad, 2005; Heuer et al., 2009).

The hopanoids, representing syn-sedimentary necrotic microbial biomass as outlined above, show in the Main Seam Fm. a ^13^C-depleted but relatively similar distributed δ^13^C_hopanoid_ signal around −30%0 (Figure 3C) compared to the bulk δ^13^C_org_ (around −26%0) (Figure 2E), pointing to a heterotrophic degradation of terrestrial OM by their source organisms (Summons et al., 2006; Talbot et al., 2016). The dominance of αβ-C_31_R-hopane is associated with bacteria known from terrestrial and more specific peat environments (Quirk et al., 1984; Huang et al., 2015; Inglis et al., 2018). In contrast, the shift to a domination of Hop-17(21)-ene and the decrease of the δ^13^C_hopanoid_ signal to values ranging between −50 and −60%0 in the Cypris Fm. (Figures 3B,C) illustrates that planktonic freshwater bacteria or benthic aerobic methanotrophs dwelling on microbially-derived methane at the water-sediment interface might have formed a significant part of the source bacteria for the deposited hopanoids (Nealson, 1997; Whiticar, 1999; Summons et al., 2006; Naeher et al., 2014; Davies et al., 2016; Hoefs, 2018).

A similar picture, resulting from the depositional environment can be seen from the depth distribution of the GDGTs. The upper Main Seam Fm. is dominated by brGDGTs characteristic for soil and swamp environments (Weijers et al., 2007, 2010; Dirghangi et al., 2013; Schouten et al., 2013; Freymond et al., 2017; Huguet et al., 2017; Naafs et al., 2017; Figure 3D). The lacustrine Cypris deposits show high but fluctuating amounts of isoGDGTs and brGDGTs representing a mixture of aquatic microbial biomass produced in the lake and terrestrial biomass from the catchment area (Weijers et al., 2007, 2010; Tierney et al., 2012; Schouten et al., 2013; Freymond et al., 2017), respectively. An aquatic *in situ* production (lacustrine or fluvial) of brGDGTs cannot be excluded, although such *in situ* productions generally seem to appear only to minor amounts (Tierney et al., 2012; DeJonge et al., 2014; Freymond et al., 2017). The distribution pattern of br-GDGT-II and –III, showing relatively similar amounts, was also investigated by Huguet et al. (2017) for bog and fen sediments and may confirm a palustrine OM input. Therefore, compared to the Main Seam Fm., the relatively higher brGDGT content might reflect a combination of *in situ* lake production and OM input from the catchment area as well as good preservation conditions within the lake sediments. Abundant isoGDGT-0 and the occurrence of archaeol and isoGDGT-1 with an initial appearance in the paleosol formation section of the Main Seam Fm. and higher occurrence in the Cypris Fm. point to the presence of methanogenic and methanotrophic archaea that have adapted from a palustrine to a lacustrine environment (Pancost et al., 2011; Dirghangi et al., 2013; Schouten et al., 2013; Bischoff et al., 2014; Naeher et al., 2014; Bale et al., 2019). In addition, an origin from archaea living in the water column cannot be excluded (Buckles et al., 2013; Schubotz et al., 2018). However, comparing the results from the hopanoid and GDGT analysis, we assume that methane cycling processes particularly of methanogenic and methanotrophic archaea and methanotrophic bacteria (hopanoid signal) played a significant role within the Early Miocene lake environment (Figure 3C).

### Deep Biosphere Structure and CO_2_-Migration Model of the Deep Hartoušov Mofette System

Our results for the present microbial community structure revealed a domination of genera from the family *Comamonadaceae* and low amounts of archaea (Figures 5D–F). Surface investigations from the Hartoušov mofette system (Beulig et al., 2015; Liu et al., 2018) and a CO_2_ vent within the Latera Caldera (Central Italy) (Oppermann et al., 2010) show relatively high abundances of acidophilic, methanogenic and iron- and sulfur-cycling microorganisms [e.g., methanogenic archaea, *Acidobacteria, Chloroflexi*, sulfate reducing bacteria (SRB), and *Geobacteraceae*]. These species either appear only to minor amounts or are completely absent in the deep subsurface sediments (Figures 5D–F), displaying the occurrence of different microbial communities in the surface and deep sections of the Hartoušov mofette system.

Considering the Early Miocene age of the investigated sediments, we suggest several community shaping selection processes during subsidence and early diagenetic transformation (Orsi et al., 2020) prior to the onset of CO_2_-migration in Mid Pleistocene. These selection processes formed a basic microbial community, which became further shaped by the ascending mantle-derived CO_2_. This assumption is based on the finding of a major microbial community composition that is in parts related to the lithological profile (Figure 6) and shows some modifications related to the occurrence of higher CO_2_ pressures and the presence of CO_2_-saturated groundwater. The major community is composed of *Actinobacteriota, Bacteroidota, Firmicutes, Patescibacteria, Alphaproteobacteria*, and dominating *Gammaproteobacteria* (Figure 5D) and appears in the Main Seam and Cypris Fm. with the difference, that the *Gammaproteobacteria* are more abundant in the Cypris Fm. (Figure 5D). A high relative abundance of *Gammaproteobacteria* and a low Shannon H index of the groundwater filter samples from the CO_2_-saturated saline aquifer seem to indicate a microbial selection and adaptation related to a high influence of ascending CO_2_-saturated groundwater, which is expressed in the upper Cypris Fm. as intercalated zones (66.7, 68.4, 70.7, and 76.2 mbs) (Figures 5C,D). As a consequence, Shannon H indices <3.2 in the lithological profile could be indicative for a high CO_2_ influence (Figure 5C).

On the genus level, the occurrence of unknown genera from the family *Comamonadaceae* in both, the groundwater and lithological samples as well as the abundance of other *Comamonadaceae* genera, namely *Acidovorax, Aquabacterium*, and *Hydrogenophaga* at the top of the Main Seam Fm., at the bottom of the Cypris Fm. and especially in the intercalated zones seem to be correlated to a mantle-derived CO_2_ influence as well (Figures 5A,E). The genera *Acidovorax* and *Hydrogenphaga* are amongst others comprised of facultative anaerobic, iron-oxidizing, and autotrophic hydrogen-oxidizing and CO_2_-fixing bacteria (Yoon et al., 2008; Byrne-Bailey et al., 2010; Willems and Gillis, 2015; Li et al., 2017). The occurrence of increased *Comamonadaceae* genera related to high CO_2_ concentrations was also reported by Ham et al. (2017), who found a predomination of *Comamonadaceae* in a natural CO_2_-dominated aquifer in South Korea as well as by Krauze et al. (2017), who detected members of *Comamonadaceae* in wet mofettes of the Cheb Basin close to the Hartoušov site. Furthermore, Mu et al. (2014) observed an increase of *Comamonadaceae* after the injection of CO_2_ into the Paaratte sandstone aquifer (Southern Australia) and a similar result was reported by Gulliver et al. (2018) for the CO_2_ injection into an aquifer at the freshwater Plant Daniel in Escatawpa (Massachusetts, USA). Although, *Comamonadaceae* have been found in non-CO_2_ influenced subsurface environments such as the Sanford Underground Research Facility (Jangir et al., 2019) and the Fennoscandian shield (Nyyssönen et al., 2014), the relative abundances in these environments were lower compared to our results. Thus, the dominance of members from the family *Comamonadaceae* in the deep sediments of the Hartoušov mofette seem to reflect a CO_2_ influenced microbial community with a good adaptation potential to the prevailing conditions. Hence, our study assumes that some members of *Comamonadaceae*, especially the determined sequences of unknown genera as well as *Acidovorax* and *Aquabacterium* are very adaptive to CO_2_-dominated ecosystems and can be suggested as indicator for such environments.

The occurrence of *Sulfuricurvum* and *Sulfurimonas* is linked to the high ${\text{SO}}_{4}^{2-}$ content of the CO_2_-saturated saline aquifer (Bussert et al., 2017) and shows an increase of *Sulfuricurvum* at the transition from the Main Seam to the Cypris Fm. and in the intercalated zones within the Cypris Fm. (Figure 5F). An abundant occurrence of the genus *Sulfuricurvum* in highly CO_2_-influenced subsurface environments was also reported by Gulliver et al. (2018). Moreover, the genus *Sulfurimonas* was found in surficial pools of several mofette systems within the Cheb Basin (Krauze et al., 2017) and in a CO_2_-driven geyser on the Colorado Plateau (Utah, USA) (Probst et al., 2018). Thus, *Sulfuricurvum* and *Sulfurimonas* might represent indicator organisms occurring in CO_2_ influenced ecosystems with a high ${\text{SO}}_{4}^{2-}$ concentration.

**Figure 8:**
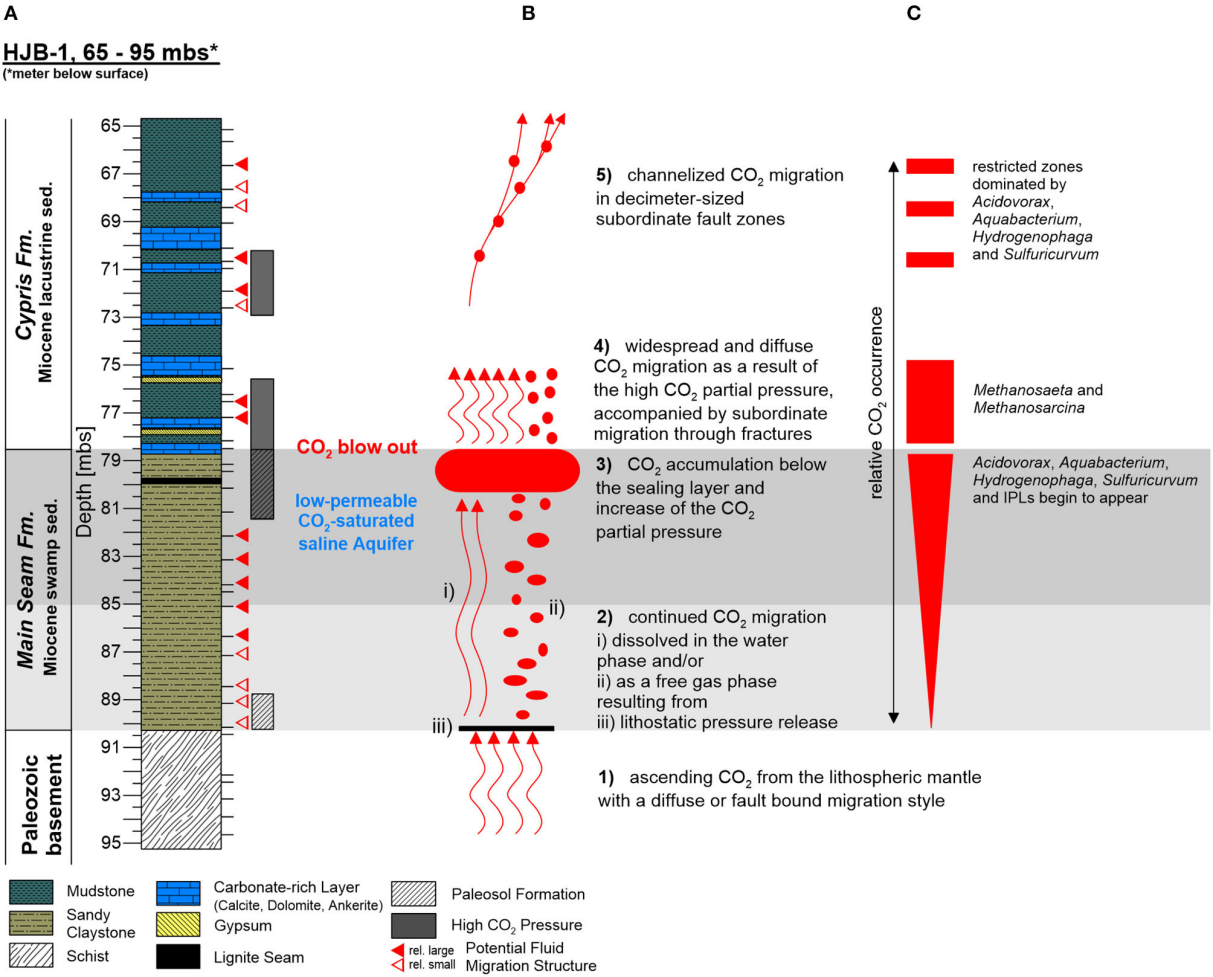
Investigated core section of the Hartoušov mofette core HJB-1 (2016) between 65 and 95 mbs showing **(A)** the stratigraphical and lithological description, **(B)** a CO_2_-migration model with the migration characteristics, and **(C)** the relative CO_2_ concentration with the distribution of CO_2_-related microbial genera.

The relationship of the investigated genera from the family *Comamonadaceae* as well as *Sulfuricurvum* and *Sulfurimonas* to the ascending CO_2_-saturated saline groundwater indicate, that the groundwater acts both, as transport mechanism and main community shaping factor for the deep biosphere. As a result, we assume the following CO_2_ migration model for the deep sediments of the Hartoušov mofette system. The CO_2_-saturated groundwater or the CO_2_ migrates from the Paleozoic basement into the low-permeable CO_2_-saturated saline aquifer and is trapped by the overlaying Cypris Fm. (Figure 8B). Thereby, related to buoyancy and the permanent CO_2_ supply from the mantle, the CO_2_ pressure increases to the top of the Main Seam Fm. with the highest concentrations occurring between 80.5 and 78.5 mbs (Figure 8C), indicated by the CO_2_ blow out during the drilling campaign (Figure 8A) and an increase in *Sulfuricurvum* and the uncommon lipid compound groups A and B (Figures 5F,G). This high CO_2_ pressure causes a widespread diffuse groundwater migration into the lower part of the Cypris Fm. (between 78.5 and 75 mbs) (Figure 8B). Therein both, potentially produced acetate from OM degradation related to a higher TOC content (Figures 2A,C) and the ascending CO_2_ itself might act as substrates for methanogenic archaea, namely *Methanosaeta*, and *Methanosarcina* (Zinder et al., 1985; Patel and Sprott, 1990; Figure 5F). Subsequently, in this core interval part of the isoGDGT signal representing methanogenic archaeal biomass (Schouten et al., 2013; Naeher et al., 2014) might also derive from the current deep biosphere (Figure 3D). This diffuse migration may also be accompanied by a subordinate migration through small fractures (Figure 8B). Afterwards, the groundwater migration seem to change into a more channelized migration (Figure 8B), indicated by the occurrence of the intercalated zones with higher abundances of *Acidovorax, Aquabacterium, Hydrogenophaga*, and *Sulfuricurvum* which correlate with fluid migration structures mentioned in the lithological description from the subsampling (73–65 mbs) (Figures 5A,E and Supplementary Figure 1C) and thus point to a CO_2_ migration related to small subordinate fault zones in decimeter-size. A possible syntrophy within this environmental setup could be based on an anaerobic, heterotrophic lifestyle of *Acidovorax, Aquabacterium*, and other genera from the family *Comamonadaceae* (Willems et al., 1990; Kalmbach et al., 1999). The fermentation of OM provides hydrogen and sulfur as substrate for the hydrogen-oxidizing *Hydrogenophaga* and the sulfur-oxidizer *Sulfuricurvum*, which both additionally utilize the ascending CO_2_ for their metabolism (Willems et al., 1989; Kodama and Watanabe, 2004).

## Conclusion

The lithological setup of the deep Hartoušov mofette system (65–95 mbs) represents a paleoenvironmental change from an Early Miocene terrestrial swamp-like (Main Seam Fm.) to a lacustrine ecosystem (Cypris Fm.). Since Mid Pleistocene time, this system became overprinted by migration and accumulation of mantle-derived CO_2_ which forms a potential habitat shaping and stimulating deep microbial life.

The necrotic microbial lipid biomarkers essentially reflect the environmental conditions during time of deposition and are therefore unsuitable for tracing the deep biosphere at the Hartoušov mofette site. This already indicates that the current biosphere signal in the deep mofette system is rather small compared to the paleo-microbial biomass.

The overall low abundance of microbial signatures from the deep biosphere in the Hartoušov mofette system suggests that the low-permeable CO_2_-saturated aquifer interval does not represent a hotspot for deep microbial life as might be expected from a substrate point of view. However, our data indicate that the availability of organic matter as microbial feedstock and CO_2_ migration are the main community shaping factors in the deep part of the mofette system. In the process, CO_2_ migration and accumulation occur heterogeneous leading to the formation of niche habitats for CO_2_-adapted microbial communities independent from the single lithological units of the explored core interval. In addition, our results imply that the high relative abundance of *Acidovorax, Aquabacterium, Hydrogenophaga*, and unknown genera of the family *Comamonadaceae* as well as the occurrence of *Sulfuricurvum* together with high sulfate contents in the CO_2_-saturated groundwater may be indicative for CO_2_-dominated deep subsurface ecosystems.

A cluster of yet unknown intact polar membrane lipids displays the presence of microbial life associated to higher accumulations of CO_2_ in the deep subsurface and show potential to act as lipid biomarkers for such environmental settings.

## Data Availability Statement

The datasets generated for this study can be found in the European Nucleotide Archive (http://www.ebi.ac.uk/ena), accession numbers PRJEB22478 (ERS4382097 to ERS4382146 and ERS4382395 to ERS4382400). Lipid data can be found in the Supplementary Material.

## Author Contributions

QL and KA wrote the manuscript, performed subsampling, and initial description of the core material as well as PCA analysis in equal manner. QL processed the geomicrobiological analysis, i.e., DNA extractions and purification, gene quantification, and bioinformatical based statistical analyses. KA performed the analysis of the intact and past lipid membrane biomarkers, and conducted together with BP the bulk elemental and bulk stable isotope analysis. FH and DL were involved in 16S rRNA sequencing data processing. PK and DL were involved in statistical analyses. DW, KM, HK, RB, and H-MS gave essential technical advice and contributed to the interpretation of the results and valuable discussion. MA and KM supervised the study and led the writing of the present manuscript. All authors have taken part in the manuscript revisions, interpretation of the results, writing of the manuscript, and agreed with its scientific content.

## Conflict of Interest

The authors declare that the research was conducted in the absence of any commercial or financial relationships that could be construed as a potential conflict of interest.
