# Supplementary material for: Microbial Signatures in Deep CO2-Saturated Miocene Sediments of the Active Hartoušov Mofette System (NW Czech Republic)
Source: Front Microbiol. 2020 Dec 14;11:543260. doi: 10.3389/fmicb.2020.543260 (PMC7768021; doi:10.3389/fmicb.2020.543260)
Supplement: Supplementary file 2 [file Data_Sheet_2.pdf]

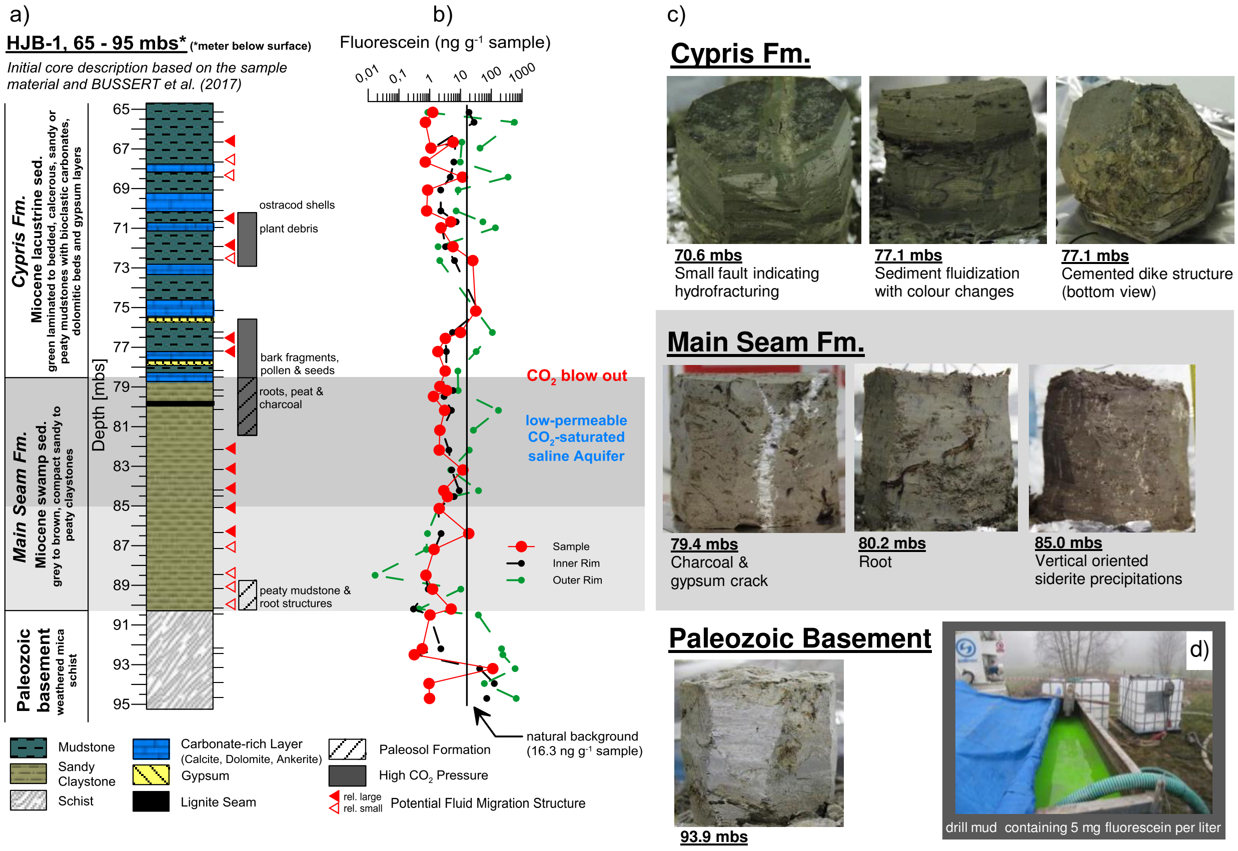

Fig. S1: Investigated core section of the Hartoušov mofette core HJB-1 (2016) between 65 and 95 mbs with depth profiles of **a)** the stratigraphical and lithological description and **b)** the determined fluorescein contents for the outer rim of the core material, the outside of the inner rim and the sample material. **c)** Exemplarily pictures of core sections from the different lithological units partly showing CO_2-fluid migration structures. **d)** Photograph from the drilling campaign showing the drill-mud container with 6m³ drill mud that was admixed with 30g of fluorescein leading to a concentration of 5 mg fluorescein per liter drill mud.

PCA results for selected lipid and genus data

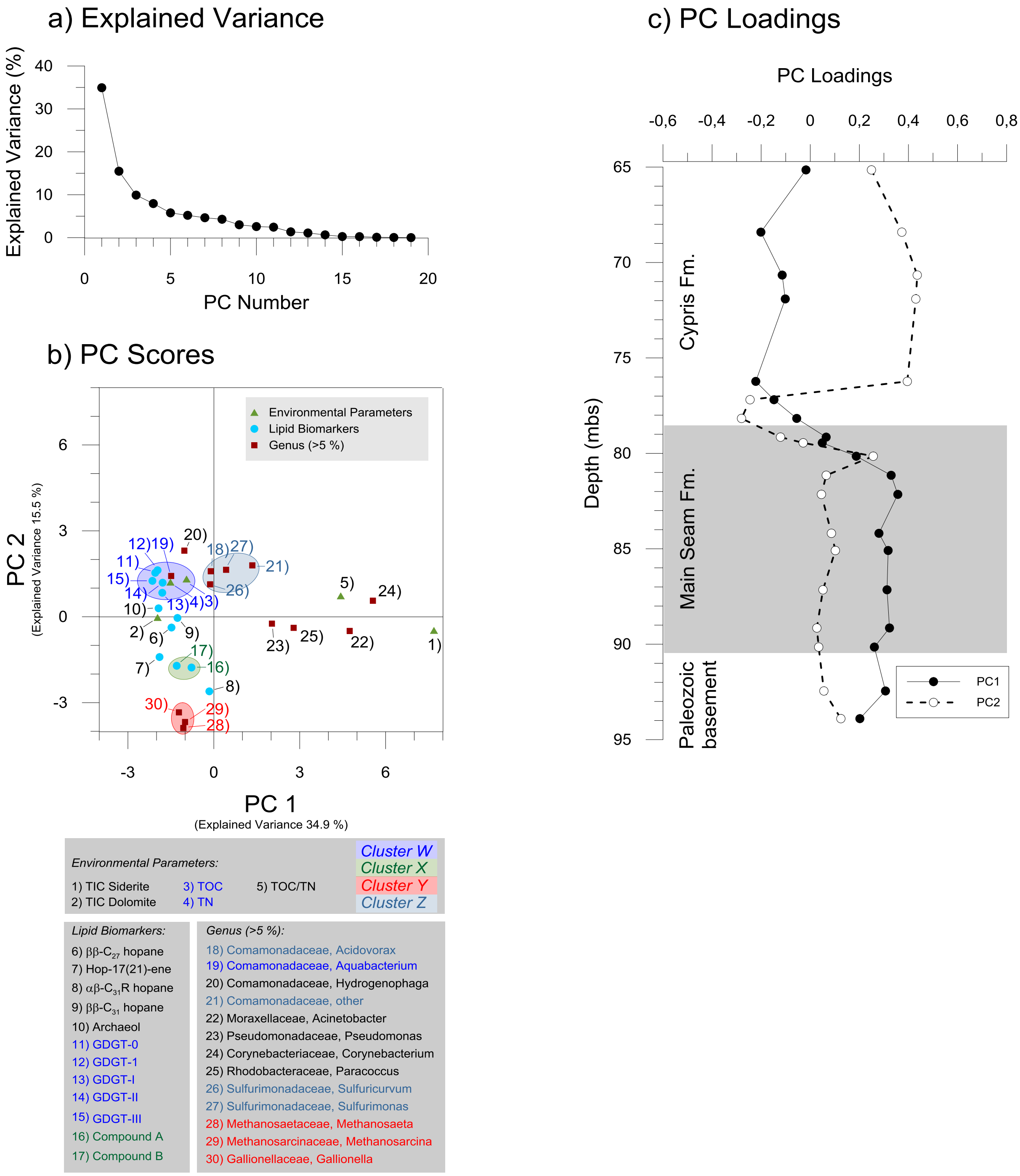

Fig. S2: Results of the principal components analysis (PCA) for selected genera and lipid biomarkers. **a)** Explained variance. **b)** Principal component (PC) scores for PC1 and PC2. **c)** PC loadings related to the lithological profile.
